# Synchrony between daily rhythms of malaria parasites and hosts is driven by an essential amino acid

**DOI:** 10.12688/wellcomeopenres.16894.2

**Published:** 2021-10-20

**Authors:** Kimberley F. Prior, Benita Middleton, Alíz T.Y. Owolabi, Mary L. Westwood, Jacob Holland, Aidan J. O'Donnell, Michael J. Blackman, Debra J. Skene, Sarah E. Reece

**Affiliations:** 1 Institute of Evolutionary Biology, University of Edinburgh, Edinburgh, UK; 2 Institute of Immunology & Infection Research, University of Edinburgh, Edinburgh, UK; 3 School of Biosciences and Medicine, University of Surrey, Surrey, UK; 4 Malaria Biochemistry Laboratory, Francis Crick Institute, London, UK; 5 Faculty of Infectious Diseases, London School of Hygiene & Tropical Medicine, London, UK

**Keywords:** Plasmodium, periodicity, circadian rhythm, metabolism, metabolomics, intraerythrocytic development cycle, isoleucine, asexual replication

## Abstract

**Background: **Rapid asexual replication of blood stage malaria parasites is responsible for the severity of disease symptoms and fuels the production of transmission forms. Here, we demonstrate that a *Plasmodium chabaudi’s* schedule for asexual replication can be orchestrated by isoleucine, a metabolite provided to the parasite in a periodic manner due to the host’s rhythmic intake of food.

**Methods: **We infect female C57BL/6 and Per1/2-null mice which have a disrupted canonical (transcription translation feedback loop, TTFL) clock with 1×10 ^5^ red blood cells containing *P. chabaudi* (DK genotype). We perturb the timing of rhythms in asexual replication and host feeding-fasting cycles to identify nutrients with rhythms that match all combinations of host and parasite rhythms. We then test whether perturbing the availability of the best candidate nutrient *in vitro* changes the schedule for asexual development.

**Results: **Our large-scale metabolomics experiment and follow up experiments reveal that only one metabolite - the amino acid isoleucine – fits criteria for a time-of-day cue used by parasites to set the schedule for replication. The response to isoleucine is a parasite strategy rather than solely the consequences of a constraint imposed by host rhythms, because unlike when parasites are deprived of other essential nutrients, they suffer no apparent costs from isoleucine withdrawal.

**Conclusions: **Overall, our data suggest parasites can use the daily rhythmicity of blood-isoleucine concentration to synchronise asexual development with the availability of isoleucine, and potentially other resources, that arrive in the blood in a periodic manner due to the host’s daily feeding-fasting cycle. Identifying both how and why parasites keep time opens avenues for interventions; interfering with the parasite’s time-keeping mechanism may stall replication, increasing the efficacy of drugs and immune responses, and could also prevent parasites from entering dormancy to tolerate drugs.

## Introduction

Circadian rhythms are assumed to have evolved to coordinate organisms’ activities with daily rhythms in the environment ( Hozer *et al.*, 2020; Ouyang *et al.*, 1998; Spoelstra *et al.*, 2016). The value of organising sleep/wake cycles according to whether it is light or dark, whether predators or prey are active, *etc*., is clear, but how parasites cope with rhythmicity in the within-host environment has been neglected ( Martinez-Bakker & Helm, 2015; Reece *et al.*, 2017; Westwood *et al.*, 2019). Many of the processes underlying interactions between hosts and parasites have a circadian basis, yet why these rhythms exist and what their consequences are for hosts and parasites remain mysterious. For example, parasites are confronted with rhythms in host behaviours and physiologies, including immune responses and metabolism. Thus, some host rhythms offer opportunities for parasites to exploit, whilst other rhythms impose constraints parasites must cope with. Some parasites use their own circadian clocks to control metabolism ( Rijo-Ferreira *et al.*, 2017), and virulence ( Hevia *et al.*, 2015), suggesting that host rhythms are a selective (evolutionary) driver of parasite rhythms. Host rhythms have fitness consequences for malaria ( *Plasmodium*) parasites ( O’Donnell *et al.*, 2011; O’Donnell *et al.*, 2013), whose rhythmicity in development during blood-stage replication is aligned with the timing of host feeding-fasting cycles ( Hirako *et al.*, 2018; O’Donnell *et al.*, 2020; Prior *et al.*, 2018).

Explaining the timing and synchrony exhibited by malaria parasites during successive cycles of blood-stage replication has been a puzzle since the Hippocratic Era, when the duration of these cycles (24-, 48, or 72-hours, depending on the *Plasmodium spp.*) were used to diagnose malaria infection ( Garcia *et al.*, 2001; Mideo *et al.*, 2013). Blood-stage asexual replication is orchestrated by the intraerythrocytic development cycle (IDC), which is characterised by parasite invasion of red blood cells (RBC), growth, division, and finally bursting of RBC to release the next cohort of asexually replicating parasites. Given that asexual replication is responsible for the disease symptoms of malaria and fuels the production of transmission forms, explaining how and why the vast majority of *Plasmodium* species progress though the IDC in synchrony, and why transitions between these stages occur at particular times of day, may unlock novel interventions and improve the efficacy of existing treatments. However, such endeavours rely on identifying the precise host rhythm(s) that parasites align to and explaining why this matters for their fitness ( Prior *et al.*, 2020).

Malaria parasites are able to keep time ( Rijo-Ferreira *et al.*, 2020; Subudhi *et al.*, 2020) and organise their IDC schedule to coordinate with an aspect(s) of host feeding-fasting rhythms ( Hirako *et al.*, 2018; Prior *et al.*, 2018), but not the act of eating itself ( Rijo-Ferreira *et al.*, 2020), or processes directly controlled by canonical host circadian clocks ( O’Donnell *et al.*, 2020). Coordination with host feeding-fasting rhythms may allow parasites to couple the nutritional needs of each IDC stage with circadian fluctuations in the concentrations of nutrients in the blood ( Prior *et al.*, 2020). Whilst parasites are able to scavenge most amino acids from haemoglobin digestion, and can biosynthesise some metabolites themselves, other nutrients are essential and must be taken up from the RBCs or blood plasma. Haemoglobin digestion and biosynthesis can occur at any time-of-day but essential nutrients that both host and parasite must acquire from the host’s food (including glucose and the amino acid, isoleucine) follow circadian rhythms in the blood ( Skene *et al.*, 2018). Hosts forage during their active phase and fast during the rest phase, which generally generates peaks in the blood concentrations of metabolites shortly after the onset of feeding and their lowest point (nadir) occurs during the fasting phase.

**Figure 1.**
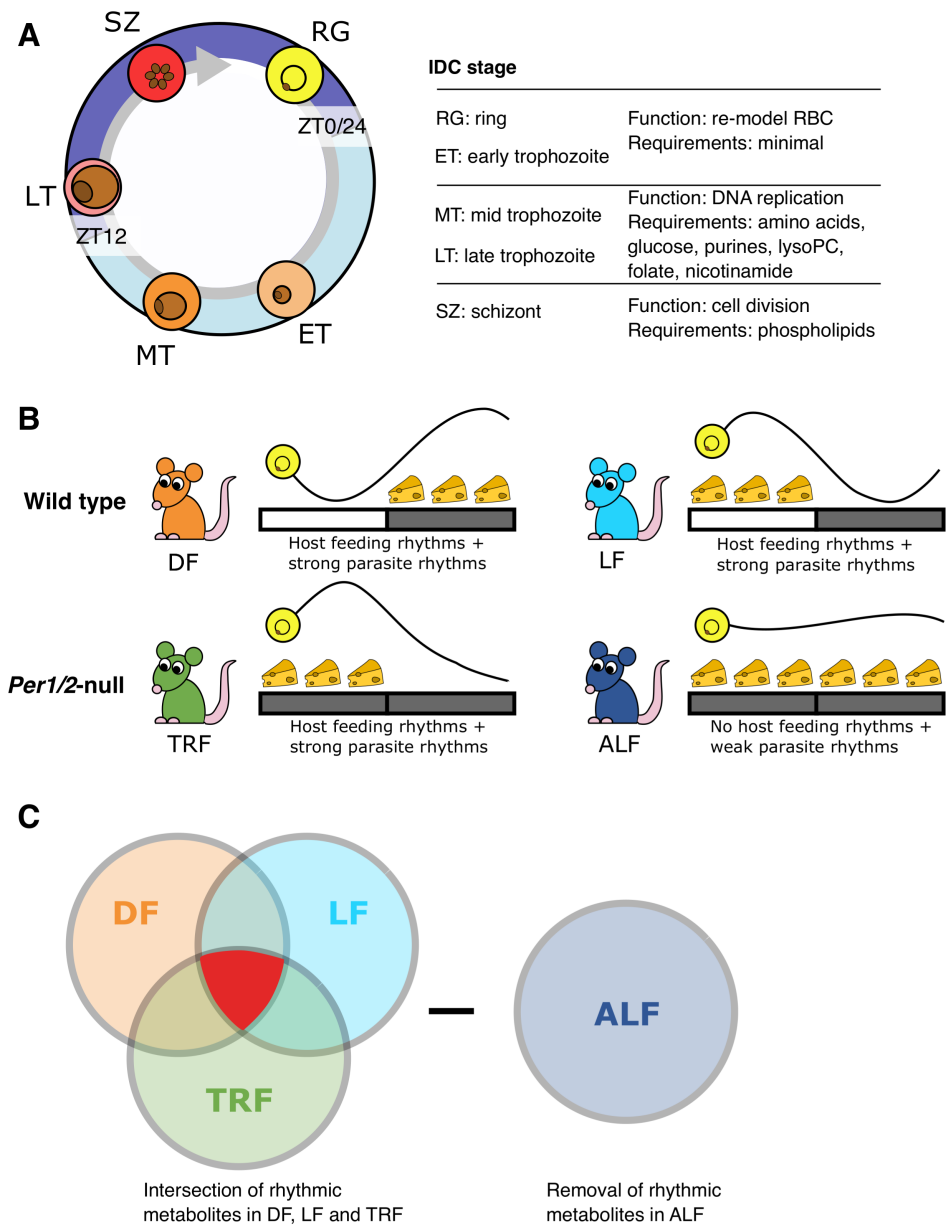
Rhythms in the intraerythrocytic development cycle (IDC) and host rhythms. ( **A**) The role of each IDC stage during cycles of asexual replication, and the resources known to be essential to each stage. RG=ring stage, ET=early stage trophozoite, MT=mid stage trophozoite, LT=late stage trophozoite, SZ=schizont. ( **B**), The experimental design used wild type mice housed in a 12h light: 12h dark regime (indicated by the light-dark bar) with unrestricted access to food for 12 hours either during the night-times (DF, dark feeding) or the day times (LF, light feeding) as indicated by the position of the cheeses. Per1/2-null mice were housed in continuous darkness (DD) and either experienced cycles of 12-hours with food followed by 12 h without access to food (TRF, time restricted feeding,) or given constant access to food (ALF, *ad libitum* feeding). The parasite IDC is rhythmic in DF, LF and TRF mice but not ALF mice ( O’Donnell *et al.*, 2020) causing the IDC rhythm to be substantially dampened. ( **C**) Metabolites that were significantly rhythmic in DF, LF and TRF mice (highlighted in red), but not in ALF mice, were sought. Treatment groups colour coded throughout: DF=orange, LF=light blue, TRF=green, ALF=dark blue.

Nutritional needs vary across IDC stages ( Figure 1A), with for example, requirements for glycerophospholipids and amino acids to fuel biogenesis, increasing as parasites progress through the IDC, and the release of parasite progeny at the end of the IDC consumes a lot of glucose ( Déchamps *et al.*, 2010; Olszewski *et al.*, 2009). Whether essential nutrients are available around-the-clock at sufficient concentrations to satisfy the needs of all parasite cells within an infection is unknown, but in culture, IDC progression is affected by nutrient limitation. For example, parasites experiencing glucose limitation quickly mount starvation responses ( Daily *et al.*, 2007) and isoleucine starvation rapidly induces dormancy ( Babbitt *et al.*, 2012). Moreover, the nadir of blood glucose rhythms in the rest phase can be exacerbated by malaria infection ( Hirako *et al.*, 2018). Thus, to avoid the problems of temporal resource limitation, we hypothesise that parasites match transitions between IDC stages to rhythmicity in the availability of essential nutrients in the blood, ensuring the most demanding IDC stages (trophozoites and schizonts) coincide with when resources are most abundant. Parasites could synchronise with resource rhythms by using an essential nutrient itself as a time-cue to set the schedule for IDC transitions, and/or allow the host to impose the IDC schedule by, for example, selectively killing IDC stages that are not “on time”. By getting the timing of the IDC schedule right, parasites garner fitness in two ways. They maximise asexual replication, which underpins within-host survival ( O’Donnell *et al.*, 2011; O’Donnell *et al.*, 2013), and benefit from coordinating the production of sexual transmission stages with the time-of-day their vectors forage for blood ( Pigeault *et al.*, 2018; Schneider *et al.*, 2018).

We integrate evolutionary ecology and parasitology with chronobiology to, first, undertake a hypothesis-driven screen of several metabolite classes and glucose to identify nutrients with daily fluctuations in the blood of malaria-infected hosts. Second, we determine which metabolites follow rhythms set by the timing of different perturbations of host feeding-fasting rhythms as well as matching the timing of the IDC schedules in all treatment groups. We find that the IDC schedule cannot be explained by fluctuations in blood glucose concentrations and almost all metabolites in the screen, but the experiment does reveal a single candidate; the amino acid, isoleucine. Third, we test if the timing of isoleucine provision and withdrawal affects IDC progression in the manner expected if parasites use it as a time-of-day cue to schedule their IDC or in the manner expected if the IDC schedule is imposed by starvation of mis-timed IDC stages. We capitalise on the rodent malaria *P. chabaudi* model system in which *in vivo* experiments exploit ecologically relevant host-parasite interactions and short-term *in vitro* studies allow within-host ecology to be probed in-depth.

## Results

### Metabolites that associate with host feeding rhythms and the IDC schedule

To identify metabolites whose rhythms correspond to the timing of host feeding and the IDC schedule, we compared four groups of malaria infections in mice that were either wild type (WT) C57BL/6J strain or Per1/2-null (Period 1 and Period 2) circadian clock-disrupted mice. Per1/2-null mice have an impaired canonical clock (transcription translation feedback loop, TTFL) and exhibit no circadian rhythms in physiology or behaviour when kept in constant darkness ( Bae *et al.*, 2001; Maywood *et al.*, 2014; O’Donnell *et al.*, 2020). We generated three different groups of hosts whose feeding-fasting rhythms differed with respect to the light:dark cycle and whether they had an intact TTFL clock, and a 4 ^th^ group of hosts which lacked both feeding rhythms and an intact TTFL clock ( Figure 1B). Specifically, we housed 2 groups of wild type mice in light-dark cycles to entrain their SCN-driven rhythms, and applied time-restricted (TRF) feeding protocols such that one group had access to food throughout the 12 hours of dark (dark fed, DF) or light (light fed, LF), every circadian cycle. Using TRF for both groups of wild types avoided any potentially confounding effects of elevated food intake on metabolite concentrations that could occur if the DF mice were instead allowed continuous access to food. Both groups of Per1/2-null mice were housed in constant darkness and one group experienced TRF such that food was available for the same daily window as the LF mice, and the other group had continuous access to food (ad lib fed, ALF). We chose these combinations because they span the manner that feeding-fasting rhythms, but not photoperiod, impact the IDC schedule, observed by us and others ( Hirako *et al*., 2018; O’Donnell *et al*., 2020; Prior *et al*., 2018; Rijo-Ferreira *et al*., 2020).

All infections were sampled every two hours from day 5 post infection (when infections are at a low parasitaemia, ~10%, to minimise the contribution of parasite molecules to the dataset, Olszewski *et al.*, 2009) for 26 hours. We hypothesised that a time-cue/time-dependent resource must vary or have rhythmicity across 24 hours, with a peak concentration that associates with the timing of host feeding-fasting across the three treatment groups with rhythmic feeding, yet also be arrhythmic in the 4 ^th^ group ( Figure 1B). In addition, the parasite IDC must be rhythmic in the three treatment groups with rhythmic feeding, with the same IDC stage present at the time of feeding, and be arrhythmic in the 4 ^th^ group. Thus, we identified candidate metabolites by intersecting rhythmic metabolites in the treatment groups ( Figure 1C).

**Figure 2.**
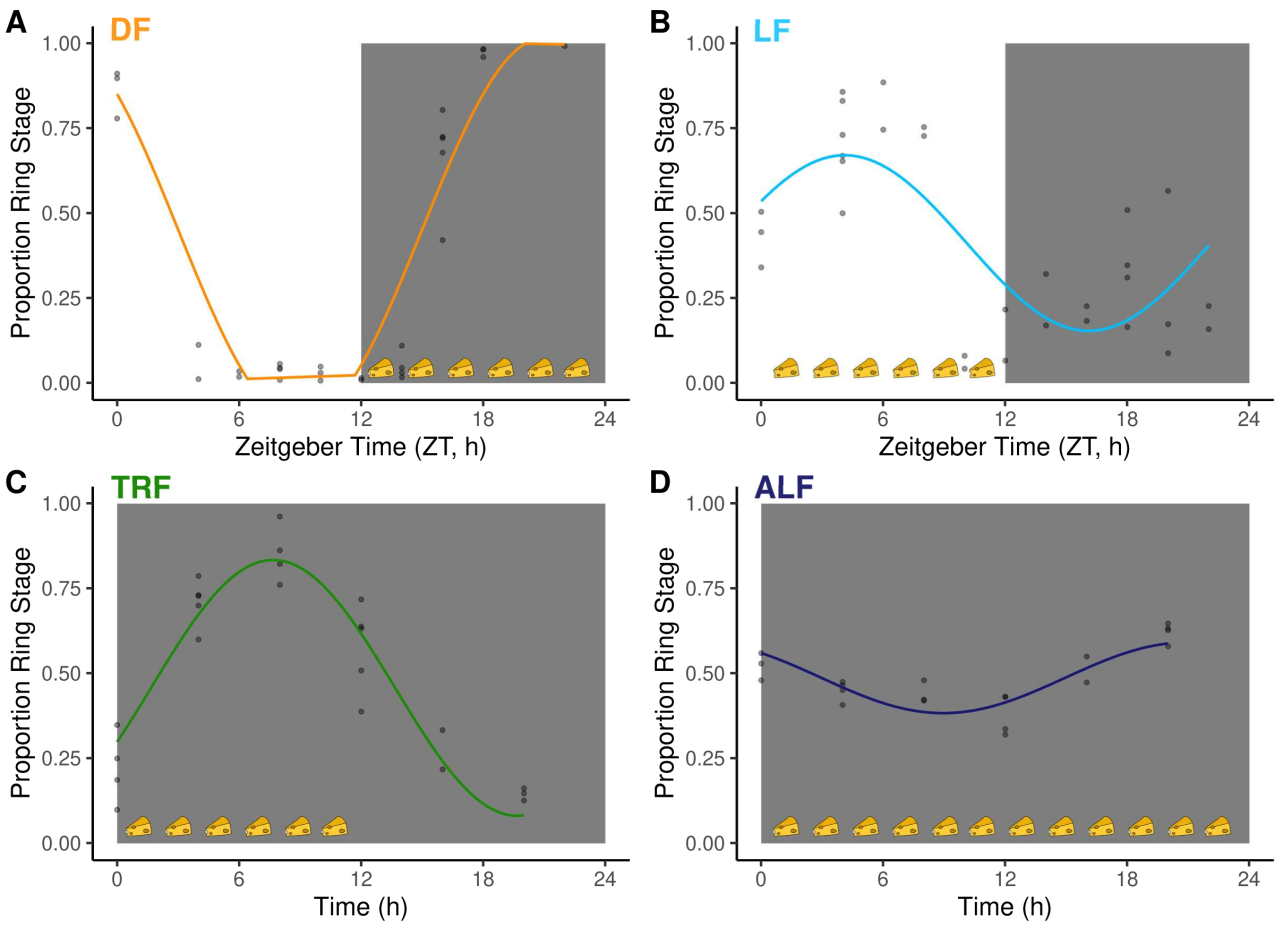
Intraerythrocytic development cycle (IDC) schedule and host feeding rhythms. The proportion of parasites at ring stage (phase marker) for: ( **A**) DF: dark feeding WT mice food access ZT 12-24 (12 h at night) in 12h:12h light:dark, ( **B**) LF: light feeding WT mice food access ZT 0-12 (12 h in day) in 12 h:12 h light:dark. ( **C**) TRF: time restricted feeding Per1/2-null mice food access 0–12 hours (12 h at the same time (GMT) as LF mice) in constant darkness (DD). ( **D**) ALF: *ad libitum* feeding Per1/2-null mice access in constant darkness (see Figure 1 for experimental design and more details). Feeding-fasting rhythms are indicated by cheeses, the white panel denotes lights on (Zeitgeber Time=0–12 h), dark grey panel denotes lights off (Zeitgeber Time=12–24 h for DF and LF, 0–24 h for TRF and ALF). Model fits for each group are plotted on the raw data (n= 2–5 infections per time point). The fitted sine/cosine curve for DF ( **A**) is distorted due to a large amplitude which exceeds the bounds possible for proportion data (between 0 and 1) so is truncated for visualisation. The patterns for groups are explained better by sine/cosine curves than by a straight line (see Table 1).

**Figure 3.**
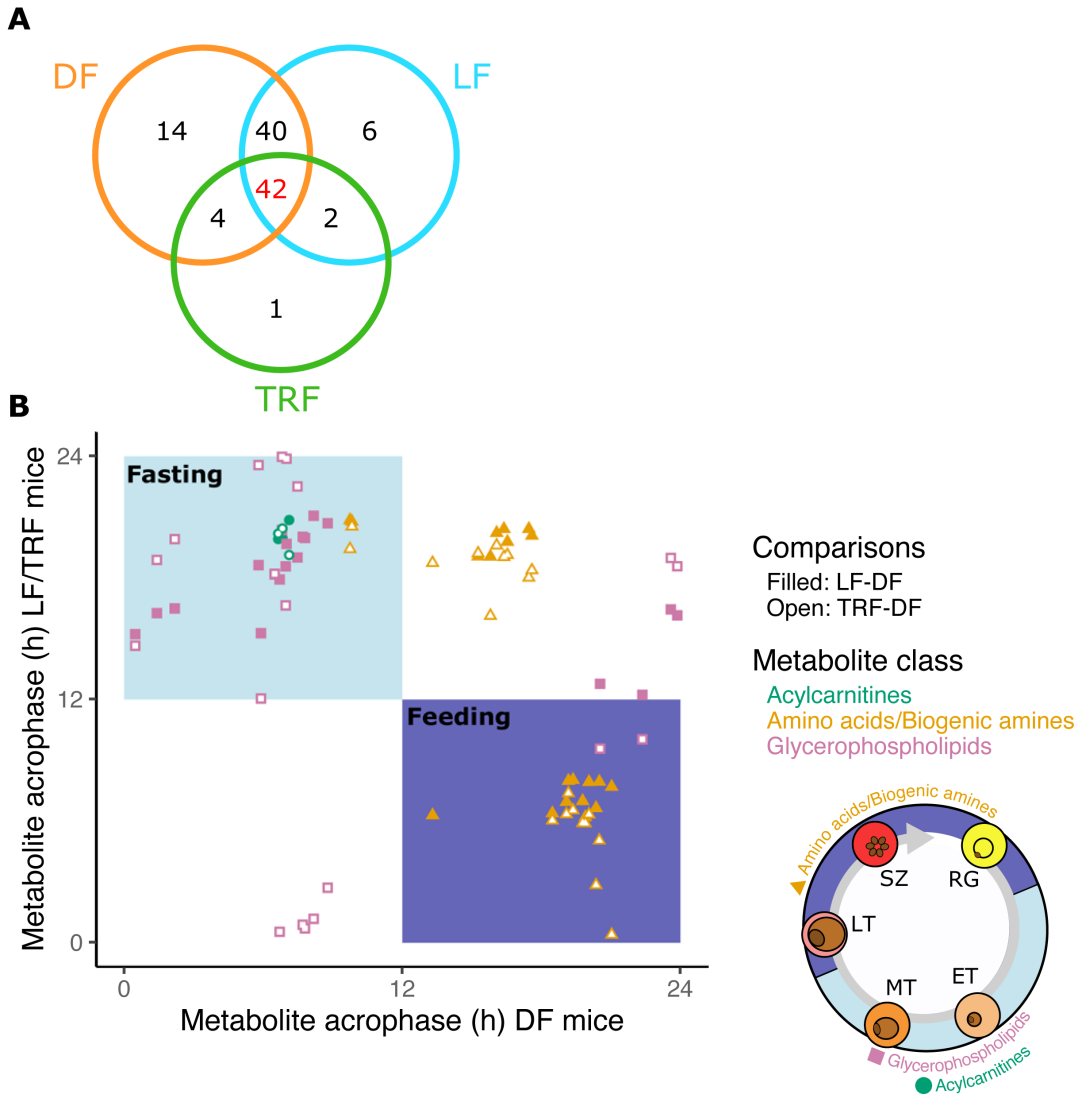
Rhythmic metabolites that associate with intraerythrocytic development cycle (IDC) timing and host feeding-fasting cycles. ( **A**) The numbers of rhythmic intersecting metabolites out of a total of 109. ( **B**) Peak timing (phase) concentration of the 42 metabolites that are rhythmic in DF, LF and TRF infections. Top left panel (light blue) denotes peaks during the fasting phase in all groups and the bottom right panel (purple) denotes peaks in the feeding phase for all groups, yielding 33 candidates linked to the feeding-fasting cycle. The top right and bottom left regions contain metabolites that are not in phase with feeding-fasting rhythms in all groups. Metabolite classes: acylcarnitines=green circles, amino acids/biogenic amines=orange triangles, glycerophospholipids=purple squares. See Table 7 for peak times of each metabolite. Ring stages (RG), late trophozoites (LT) and schizonts (SZ) peak during the hosts feeding period (purple) when some amino acids/biogenic amines peak. Early trophozoites (ET) and mid trophozoites (MT) peak during host fasting (light blue) when glycerophospholipids and acylcarnitines peak.

IDC schedules followed the expected patterns for each treatment group ( Figure 2, and O’Donnell *et al.*, 2020). Specifically, parasites displayed opposite IDC schedules in dark (i.e. night, DF, n=18) and light (i.e. day, LF, n=17) fed WT mice because their feeding-fasting rhythms are inverted ( Figure 2, Table 1). Parasites in Per1/2-null mice (kept in constant darkness), fed during a time-restricted 12 h window (TRF mice, n=17; Table 2) that coincided with when LF mice were fed, followed the same IDC schedule as parasites in LF mice. Finally, parasites in Per1/2-null mice (kept in constant darkness) with constant access to food (ALF mice, n=16; Table 2) exhibited dampened IDC rhythms because their hosts have no feeding-fasting rhythm ( O’Donnell *et al.*, 2020). Across the entire data set, 110 metabolites exhibited variation in their blood concentrations during the 26-hour sampling window (101 in DF, 91 in LF, 50 in TRF and one in ALF; Table 3). That only one metabolite (lysoPC a C20:3) exhibited a rhythm in ALF hosts demonstrates that host TTFL clocks and timed feeding are required to generate metabolite rhythms ( Figure 3A, Reinke & Asher, 2019). Further, that approximately half of the metabolites rhythmic in hosts with TTFL clocks (DF and LF) were also rhythmic in TRF hosts reveals that feeding rhythms alone can maintain metabolic rhythms during infection ( Figure 3A). Of all metabolites, only 42 were rhythmic in all three groups of infections with both feeding-fasting and IDC rhythms (i.e. the red area in Figure 1C), consisting of three acylcarnitines, 11 amino acids, nine biogenic amines and 19 glycerophospholipids ( Figure 3A). Next, we identified 33 metabolites that exhibited a peak concentration (timing or phase) that corresponded to the timing of the host’s feeding-fasting rhythm and the parasite’s IDC ( Figure 3B; metabolites clustering in the light blue and purple areas).

**Table 1.** Degrees of Freedom (df), log-Likelihood (logLik), AICc, ΔAICc (AICc _i_ ΔAICc _min_) and AICc w (AICc weight) for each linear model in the parasite stage proportion analysis ordered in descending fit (best-fitting model at the top). The response variable for each model is proportion of ring stages (Ring.prop), with “sine” and “cosine” terms being the sine or cosine function of (2π × time of day)/24 with a fixed 24-hour period fitted for each treatment group (DF, LF, TRF or ALF). AICc is a form of the Akaike Information Criteria corrected for smaller sample sizes to address potential overfitting, used for model selection. DF=dark fed wild type mice, LF=light fed wild type mice, TRF=time restricted fed Per1/2-null mice, ALF=ad libitum fed Per1/2-null mice.

	Model description: Ring.prop ~	df	logLik	AICc	ΔAICc	AICc *w*
DF	sine + cosine	4	13.30	-16.9	0.00	1.000
	sine	3	-5.77	18.5	35.44	0.000
	cosine	3	-8.69	24.3	41.27	0.000
	null	2	-14.60	33.7	50.60	0.000
LF	sine + cosine	4	8.57	-7.5	0.00	0.788
	sine	3	5.92	-4.9	2.63	0.212
	cosine	3	-1.03	9.0	16.52	0.000
	null	2	-2.68	9.8	17.34	0.000
TRF	sine + cosine	4	21.85	-33.5	0.00	1.000
	sine	3	10.98	-14.7	18.77	0.000
	cosine	3	-0.14	7.5	41.01	0.000
	null	2	-2.54	9.7	43.15	0.000
ALF	sine + cosine	4	32.12	-53.6	0.00	0.995
	sine	3	24.51	-41.5	12.05	0.002
	cosine	3	24.49	-41.5	12.08	0.002
	null	2	19.90	-35.1	18.47	0.000

**Table 2.** Mouse numbers for experimental treatment groups. A) Mice in the metabolomics experiment were sampled in blocks (A–D – see methods) with 4/5 mice per block sampled every eight hours. Totals for each treatment group: DF=18, LF=17, TRF=17, ALF=16. B) Mice in the glucose experiment were sampled every two hours. Totals for each treatment group: DF=5, LF=5, TRF=5, ALF=5. For each experiment repeated measures from mice were controlled for during the analysis. DF=dark fed wild type mice, LF=light fed wild type mice, TRF=time restricted fed Per1/2-null mice, ALF=ad libitum fed Per1/2-null mice.

Time (ZT/hour)	0	2	4	6	8	10	12	14	16	18	20	22	24/0	2
**A** Metabolomics experiment														
*Block*	*A*	*B*	*C*	*D*	*A*	*B*	*C*	*D*	*A*	*B*	*C*	*D*	*A*	*B*
DF	5	5	4	4	5	5	4	4	5	5	4	4	5	5
LF	5	4	4	4	5	4	4	4	5	4	4	4	5	4
TRF	5	4	4	4	5	4	4	4	5	4	4	4	5	4
ALF	4	4	4	4	4	4	4	4	4	4	4	4	4	4
**B** Glucose experiment														
DF	5	5	5	5	5	5	5	5	5	5	5	5	5	5
LF	5	5	5	5	5	5	5	5	5	5	5	5	5	5
TRF	5	5	5	5	5	5	5	5	5	5	5	5	5	5
ALF	5	5	5	5	5	5	5	5	5	5	5	5	5	5

**Table 3.** Metabolite numbers that significantly fluctuate every 24 hours in the mouse blood for three methods. **A**) Data for all metabolites were run through three circadian programmes to find those following a 24h rhythm using ECHO (Benjamini-Hochberg (BH) adjusted p value of 0.05), CircWave (standard p value of 0.05) and JTK_Cycle (BH adjusted p value of 0.05). Metabolites that are excluded using the Surrey LC/MS assay criteria were removed from the analysis ( Figure 10). **B**) Rhythmic metabolites in each programme (ECHO, CircWave and JTK) were intersected, with a metabolite counted as rhythmic if it is significantly rhythmic in at least two programmes (ECHO=CW, ECHO=JTK, CW=JTK). **C**) The metabolites not fulfilling these criteria were analysed using ANOVA (including time-of-day as a factor), with BH adjusted p values at the 5% level. Metabolites from both methods (circadian programmes and ANOVA) were then combined to perform a final intersection between DF, LF and TRF to find common metabolites. Metabolites rhythmic in ALF mice were then removed. DF=dark fed wild type mice, LF=light fed wild type mice, TRF=time restricted fed Per1/2-null mice, ALF=ad libitum fed Per1/2-null mice.

A	ECHO		CircWave (CW)		JTK	
	Rhythmic with 24h period	Not-rhythmic with 24h period	Rhythmic with 24h period	Not-rhythmic with 24h period	Rhythmic with 24h period	Not-rhythmic with 24h period
DF	75	59	67	67	47	87
LF	106	28	83	51	70	64
TRF	55	77	41	93	23	111
ALF	2	127	6	128	0	134
**B**	ECHO==CW	ECHO==JTK	CW==JTK		Unique	
DF	62	47	46		63	
LF	79	68	63		88	
TRF	37	23	21		39	
ALF	1	0	0		1	
**C**	Not-rhythmic with 24h period	Time-of-day effect	No time-of- day effect	Time-of-day effect + rhythmic with 24h period	Total candidates	
DF	71	38	33	101	42	
LF	46	3	43	91		
TRF	95	11	84	50		
ALF	133	0	133	1		

### Glucose does not associate with the IDC schedule

**Figure 4.**
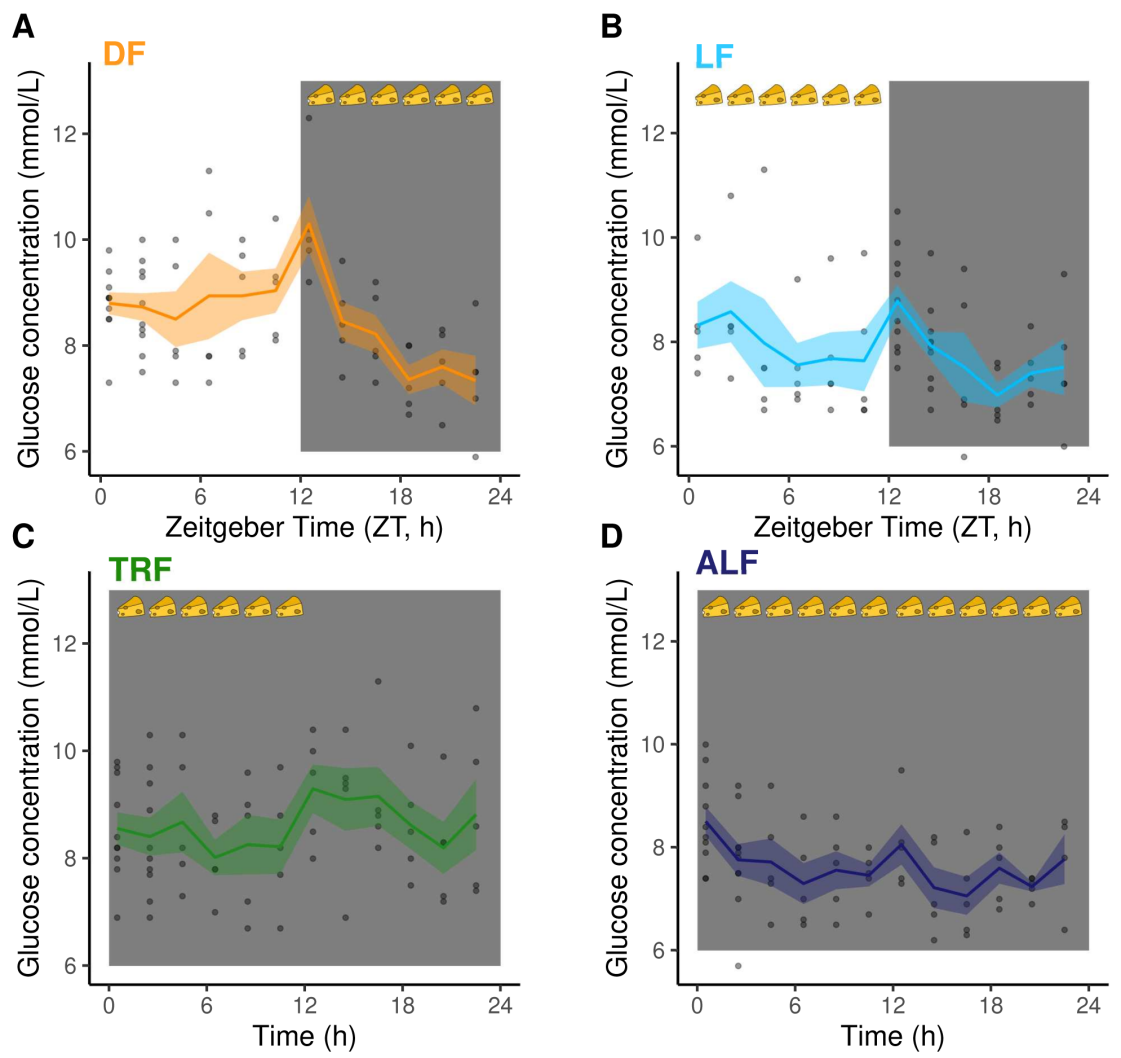
Blood glucose concentrations (mmol/L). ( **A**) DF: dark feeding WT mice food access ZT 12-24 (12 h at night) in 12h:12h light:dark, ( **B**) LF: light feeding WT mice food access ZT 0-12 (12 h in day) in 12 h:12 h light:dark. ( **C**) TRF: time restricted feeding Per1/2-null mice food access 0–12 hours (12 h at the same time (GMT) as LF mice) in constant darkness (DD). ( **D**) ALF: *ad libitum* feeding Per1/2-null mice access in constant darkness (see Figure 1 for experimental design). Feeding-fasting rhythms are indicated by cheeses, the white panel denotes lights on (Zeitgeber Time=0–12 h), dark grey panel denotes lights off (Zeitgeber Time=12–24 h for DF and LF, 0–24 h for TRF and ALF). The lines and shading are mean ± SEM at each time point and the dots are the raw data. At time points 0.5 and 2.5 for DF, TRF and ALF, and 12.5 and 14.5 for LF the points are stacked because the time course lasted ~26 hours and the data are plotted on a 0–24 hour axis.

Previous work suggests that blood glucose rhythms are responsible for the IDC schedule ( Hirako *et al.*, 2018; Prior *et al.*, 2018) but glucose could not be measured by the UPLC/MS-MS metabolomics platform. Therefore, we set up an additional set of DF, LF, TRF and ALF infections (n=5/group), which followed the same feeding-fasting and IDC schedules as infections in the metabolomics screen, Figure 2, Table 2), to quantify blood-glucose rhythms. Mean blood-glucose concentration across the time series differed between the groups, being higher in DF and TRF mice (DF=8.55±0.14 mmol/L, TRF=8.59±0.13 mmol/L) than in LF and ALF mice (LF=7.90±0.14 mmol/L, ALF=7.68±0.11 mmol/L). We found that two competitive models, including only time-of-day (Zeitgeber time, ZT/h) or both time-of-day and treatment (DF, LF, TRF and ALF) as main effects, can explain patterns of glucose concentration ( Figure 4, Table 4). Specifically, glucose concentration varied throughout the day in DF mice but much less in LF mice, and glucose was invariant in both groups of Per1/2-null mice (TRF and ALF) ( Table 4). The rhythmic IDC schedule in the DF, LF and TRF groups but the lack of significant rhythmicity in blood glucose in the TRF (and possibly LF) infections indicates that glucose does not explain the connection between feeding rhythms and the IDC schedule across all groups.

**Table 4.** Degrees of Freedom (df), log-Likelihood (logLik), AICc, ΔAICc (AICc _i_ – AICc _min_) and AICc w (AICc weight) for each linear model in the glucose concentration analysis ordered in descending fit (best-fitting model at the top). The response variable for each model is glucose concentration (Glucose.conc) and the random effect is “mouseID”. “Treatment” refers to the treatment group (DF, LF, TRF or ALF) and “time” refers to the time-of day (ZT 0-24h or 0-24h) which was fitted as a factor. DF=dark fed wild type mice, LF=light fed wild type mice, TRF=time restricted fed Per1/2-null mice, ALF=ad libitum fed Per1/2-null mice.

	Model description: Glucose.conc ~ + (1|mouseID)	df	logLik	AICc	ΔAICc	AICc *w*
	time	14	-369.71	769.0	0.00	0.555
	treatment + time	17	-366.55	769.4	0.44	0.445
	treatment*time	50	-331.38	785.1	16.12	0.000
	null	3	-390.80	787.7	18.68	0.000
	treatment	6	-387.75	787.8	18.80	0.000
**DF**	time	14	-90.06	215.7	0.00	0.996
	null	3	-110.28	226.9	11.17	0.004
**LF**	null	3	-94.83	196.0	0.00	0.550
	time	14	-80.39	196.4	0.40	0.450
**TRF**	null	3	-85.23	176.8	0.00	0.999
	time	14	-77.54	190.7	13.9	0.001
**ALF**	null	3	-88.02	182.4	0.00	0.994
	time	14	-78.43	192.6	10.21	0.006

Further, we directly tested whether glucose can schedule the IDC by comparing IDC rhythms in Per1/2-null mice (housed in DD) experiencing daily transient perturbations to blood glucose concentration. At the same time each day, infected mice (n= 5 or 6 / group) experienced one of the following treatments for 6 days: intra-peritoneal injection of glucose alone (to simulate the effects of a large meal) or with insulin (to reduce blood glucose, following Crosby *et al*., 2019), or PBS carrier only (control). Mice were infected with synchronous ring stages from WT donors, injections began on day 1 post infection and occurred 12 hours out of phase to peak feeding in the donor mice. Thus, the only rhythm parasites were exposed to was a predictable daily rhythm in blood glucose concentration. We reasoned that if parasites responded to a spike or trough in blood glucose, the IDC schedule would diverge from the controls in the glucose and/or the glucose+insulin groups.

**Figure 5.**
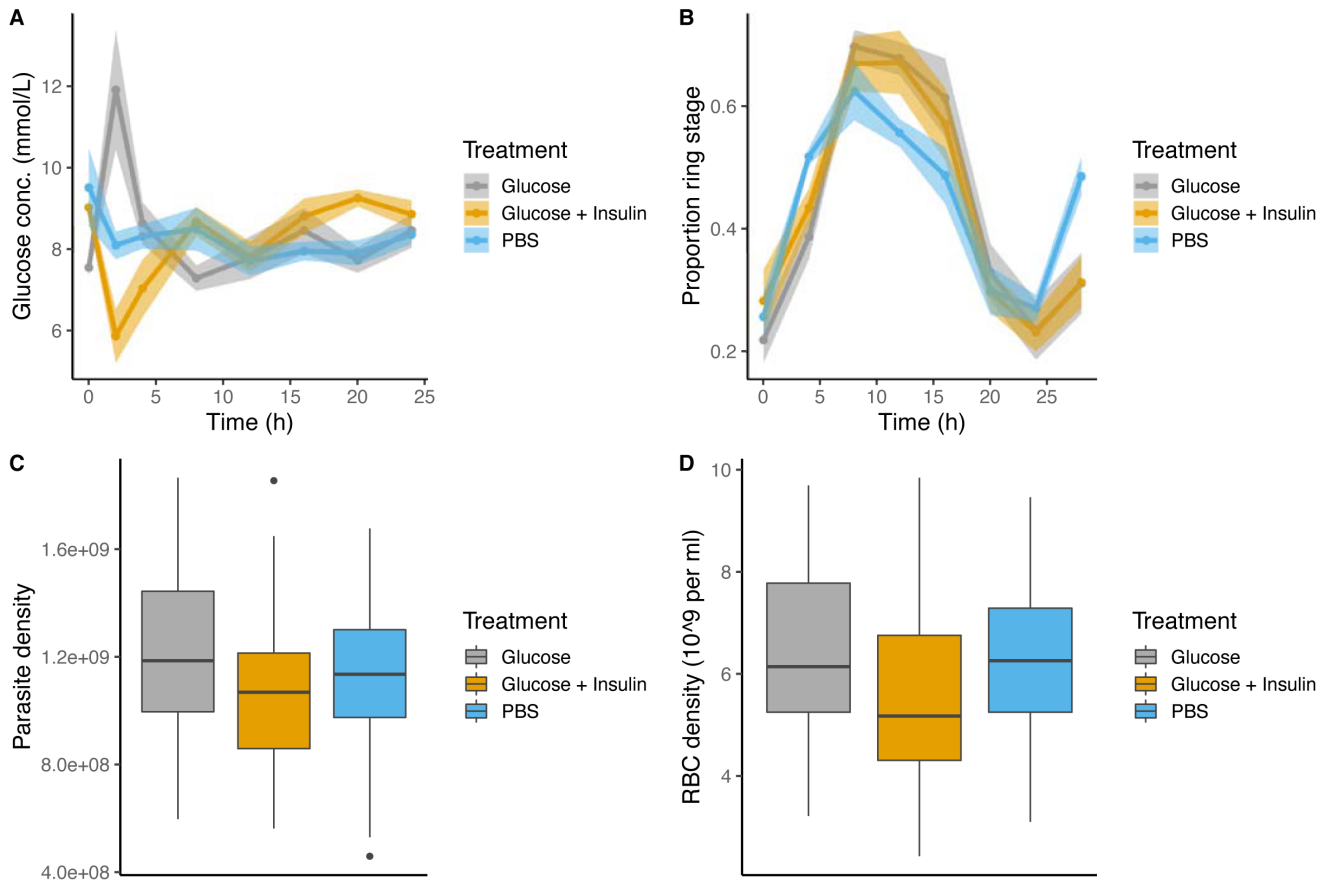
Predictable daily perturbations of blood glucose concentration do not influence the IDC schedule nor the density of parasites or red blood cells. For 6 days following infection **A**) Mice were injected at Time=1h with either glucose (grey, n=5), glucose + insulin (yellow, n=6) or PBS (n=5) and their glucose concentration (mmol/L) measured in the blood every 4 hours for 24 hours over days 5–6 post infection. **B**) Over the same 24 hour window on days 5–6 the proportion of ring stage parasites was quantified as a marker for the IDC schedule. **C**) Parasite and **D**) red blood cell density across the 24 sampling window for each treatment. Means and ±SEM plotted.

After 5 days we quantified blood glucose concentration and the IDC schedule every 4 hours for 24 hours, starting 1 hour post injections. We found glucose concentration increases or decreases, as expected, with the best fitting model including the effect of “time” and “treatment” plus their interaction (ΔAICc=0, Figure 5A, Table 5A). Specifically, blood glucose concentration remained stable in control mice that received daily injections of PBS (8.30±0.17 mmol/L). Whereas, compared to the control group, the glucose only treatment experienced elevated blood glucose (by 3.62 mmol/L) and the insulin + glucose group experienced reduced blood glucose (by 2.42 mmol/L). Perturbations of blood glucose were transient, returning to the levels of control mice 2–4 hours post injection. Despite regularly perturbing glucose levels over a sufficient number of days for the IDC schedule to change, we find no effect on parasite rhythms, with all groups following near identical rhythms ( Figure 5B, Table 5B). The best fitting model included only the effect of “time” (ΔAICc=0); the addition of “treatment” did not improve the model fit (ΔAICc=4.72). Furthermore, parasite or RBC density did not vary significantly between the treatment groups ( Figure 5C and 5D, Table 5C and 5D) suggesting parasite replication activities are impervious to time of day signals provided by glucose.

**Table 5.** Degrees of Freedom (df), log-Likelihood (logLik), AICc, ∆AIC (AICci – AICcmin) and AICc w (AICc weight) for each linear model ordered in descending fit (best-fitting model at the top) for glucose perturbations. The response variable for each model is either proportion of ring stage parasites (Ring.prop), parasite density (Parasite) or RBC density (RBC).

Model description:	df	logLik	AICc	∆AIC	AICc *w*
A) Glucose.conc ~ + (1|mouseID)					
hour * treatment	26	-184.802	435.6	0.00	1
treatment	5	-225.597	461.7	26.05	0
null	3	-230.388	467.0	31.33	0
hour + treatment	12	-221.788	470.3	34.67	0
hour	10	-226.544	475.0	39.34	0
B) Ring prop ~					
hour	9	115.438	-211.4	0.00	0.911
hour + treatment	11	115.456	-206.6	4.72	0.086
hour * treatment	25	131.257	-199.8	11.58	0.003
null	2	33.888	-63.7	147.67	0.000
treatment	4	33.893	-59.5	151.89	0.000
C) Parasite ~ + (1|hour) + (1|mouseID)					
null	4	-7.358	23.0	0.00	0.98
treatment	6	-9.053	30.8	7.76	0.02
D) RBC ~ + (1|hour) + (1|mouseID)					
null	4	224.77	-441.2	0.00	0.999
treatment	6	219.69	-426.7	14.54	0.001

### Linking metabolites to the IDC schedule

**Figure 6.**
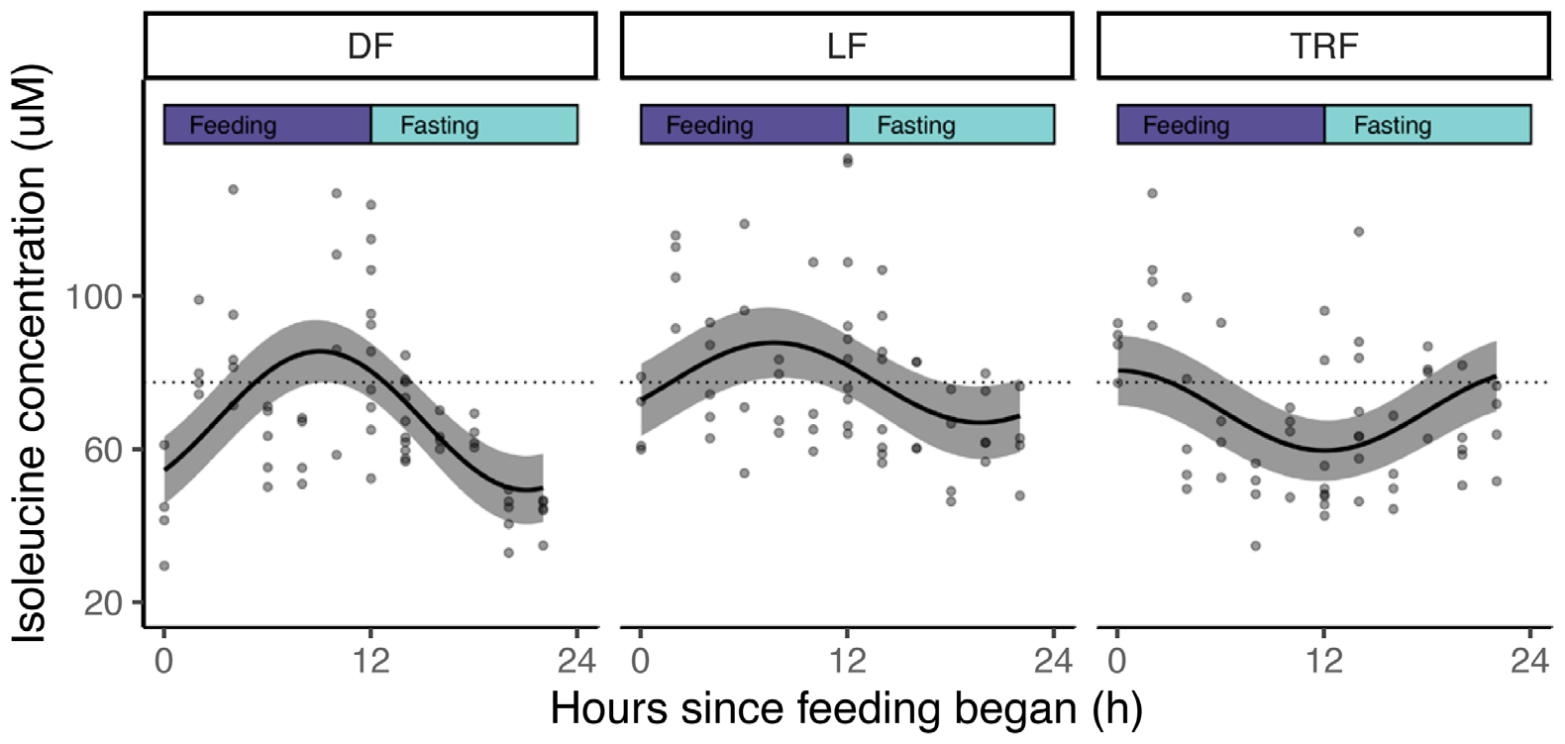
Isoleucine is rhythmic and coincides with the intraerythrocytic development cycle (IDC) schedule and host feeding-fasting cycles. Model fit (best fit line and 95% prediction interval) for DF, LF and TRF infections, from the time since feeding commences, with feeding-fasting windows overlaid. In the ALF group, a flat line with an intercept of 77.7 μM (illustrated as a dotted line) is the best fit to describe isoleucine concentration across the sampling window.

Having ruled out a role for blood glucose, we explored whether the metabolites from our screen could explain why the IDC schedule follows feeding-fasting rhythms. The metabolites whose peak associated with both feeding and late IDC stages, are alanine, asparagine, isoleucine, leucine, methionine, phenylalanine, proline, threonine, valine, methionine-sulfoxide and serotonin. Whereas 20 acylcarnitines and glycerophospholipids (with the exception of two biogenic amines) associated with fasting and early IDC stages (ADMA, SDMA, C14.1, C16, C18.1, lysoPCaC16:1, lysoPCaC18:1, lysoPCaC18:2, PCaaC32:1, PCaaC34:4, PCaaC38:3, PCaaC38:4, PCaaC38:5, PCaaC38:6, PCaaC40:4, PCaaC40:5, PCaeC34:3, PCaeC38:0, PCaeC38:3 and PCaeC42:1; Table 6 and Table 7). We asked if these metabolites could schedule the IDC through the following non-mutually exclusive mechanisms. First, by being sufficiently limiting at a certain time-of-day to enforce a rhythm on the IDC because mis-timed IDC stages starve and die. This scenario requires that parasites are unable to overcome resource limitation by synthesising the metabolite itself or scavenging it from a source (such as haemoglobin) that is available around the clock. Second, by acting as a time-of-day cue to which certain IDC stages respond by for example, initiating the transition to the next stage (called a “just-in-time” strategy) or by acting as a Zeitgeber to entrain an endogenous circadian clock that allows parasites to anticipate when IDC transitions should occur. For a metabolite to be a reliable time-of-day cue/Zeitgeber, it should be something the parasite cannot synthesise/scavenge to avoid the challenge of differentiating between inaccurate endogenous and accurate exogenous time information leading to the risk of mistakenly responding to an endogenous signal. Of the 33 metabolites on the shortlist, only isoleucine fulfils these criteria ( Table 8, Figure 6), suggesting that parasites could use isoleucine both as a resource and a time-of-day cue.

**Table 6.** Highlighted in green are the metabolites rhythmic in each treatment group (according to the circadian programmes and ANOVA). DF: 101 y, 33 n; LF: 91 y, 43 n; TRF: 50 y, 84 n; ALF: 1 y, 133 n.

Metabolite	Class	DF	LF	TRF	ALF
Ala	Amino.acid				
Arg	Amino.acid				
Asn	Amino.acid				
Asp	Amino.acid				
Cit	Amino.acid				
Gln	Amino.acid				
Glu	Amino.acid				
Gly	Amino.acid				
His	Amino.acid				
Ile	Amino.acid				
Leu	Amino.acid				
Lys	Amino.acid				
Met	Amino.acid				
Orn	Amino.acid				
Phe	Amino.acid				
Pro	Amino.acid				
Ser	Amino.acid				
Thr	Amino.acid				
Trp	Amino.acid				
Tyr	Amino.acid				
Val	Amino.acid				
ADMA	Biogenic_amine				
alpha.AAA	Biogenic_amine				
Carnosine	Biogenic_amine				
Histamine	Biogenic_amine				
Kynurenine	Biogenic_amine				
Met.SO	Biogenic_amine				
Putrescine	Biogenic_amine				
Sarcosine	Biogenic_amine				
SDMA	Biogenic_amine				
Serotonin	Biogenic_amine				
Spermidine	Biogenic_amine				
t4.OH.Pro	Biogenic_amine				
Taurine	Biogenic_amine				
C0	Acylcarnitines				
C2	Acylcarnitines				
C3	Acylcarnitines				
C4	Acylcarnitines				
C14.1	Acylcarnitines				
C16	Acylcarnitines				
C18.1	Acylcarnitines				
lysoPC.a.C16.0	Glycerophospholipids				
lysoPC.a.C16.1	Glycerophospholipids				
lysoPC.a.C17.0	Glycerophospholipids				
lysoPC.a.C18.0	Glycerophospholipids				
lysoPC.a.C18.1	Glycerophospholipids				
lysoPC.a.C18.2	Glycerophospholipids				
lysoPC.a.C20.3	Glycerophospholipids				
lysoPC.a.C20.4	Glycerophospholipids				
lysoPC.a.C24.0	Glycerophospholipids				
lysoPC.a.C26.0	Glycerophospholipids				
lysoPC.a.C26.1	Glycerophospholipids				
lysoPC.a.C28.0	Glycerophospholipids				
lysoPC.a.C28.1	Glycerophospholipids				
PC.aa.C24.0	Glycerophospholipids				
PC.aa.C28.1	Glycerophospholipids				
PC.aa.C30.0	Glycerophospholipids				
PC.aa.C32.0	Glycerophospholipids				
PC.aa.C32.1	Glycerophospholipids				
PC.aa.C32.2	Glycerophospholipids				
PC.aa.C32.3	Glycerophospholipids				
PC.aa.C34.1	Glycerophospholipids				
PC.aa.C34.2	Glycerophospholipids				
PC.aa.C34.3	Glycerophospholipids				
PC.aa.C34.4	Glycerophospholipids				
PC.aa.C36.1	Glycerophospholipids				
PC.aa.C36.2	Glycerophospholipids				
PC.aa.C36.3	Glycerophospholipids				
PC.aa.C36.4	Glycerophospholipids				
PC.aa.C36.5	Glycerophospholipids				
PC.aa.C36.6	Glycerophospholipids				
PC.aa.C38.0	Glycerophospholipids				
PC.aa.C38.3	Glycerophospholipids				
PC.aa.C38.4	Glycerophospholipids				
PC.aa.C38.5	Glycerophospholipids				
PC.aa.C38.6	Glycerophospholipids				
PC.aa.C40.2	Glycerophospholipids				
PC.aa.C40.3	Glycerophospholipids				
PC.aa.C40.4	Glycerophospholipids				
PC.aa.C40.5	Glycerophospholipids				
PC.aa.C40.6	Glycerophospholipids				
PC.aa.C42.0	Glycerophospholipids				
PC.aa.C42.1	Glycerophospholipids				
PC.aa.C42.2	Glycerophospholipids				
PC.aa.C42.4	Glycerophospholipids				
PC.aa.C42.5	Glycerophospholipids				
PC.aa.C42.6	Glycerophospholipids				
PC.ae.C30.1	Glycerophospholipids				
PC.ae.C30.2	Glycerophospholipids				
PC.ae.C32.1	Glycerophospholipids				
PC.ae.C32.2	Glycerophospholipids				
PC.ae.C34.0	Glycerophospholipids				
PC.ae.C34.1	Glycerophospholipids				
PC.ae.C34.2	Glycerophospholipids				
PC.ae.C34.3	Glycerophospholipids				
PC.ae.C36.0	Glycerophospholipids				
PC.ae.C36.1	Glycerophospholipids				
PC.ae.C36.2	Glycerophospholipids				
PC.ae.C36.3	Glycerophospholipids				
PC.ae.C36.4	Glycerophospholipids				
PC.ae.C36.5	Glycerophospholipids				
PC.ae.C38.0	Glycerophospholipids				
PC.ae.C38.1	Glycerophospholipids				
PC.ae.C38.2	Glycerophospholipids				
PC.ae.C38.3	Glycerophospholipids				
PC.ae.C38.4	Glycerophospholipids				
PC.ae.C38.5	Glycerophospholipids				
PC.ae.C38.6	Glycerophospholipids				
PC.ae.C40.1	Glycerophospholipids				
PC.ae.C40.2	Glycerophospholipids				
PC.ae.C40.3	Glycerophospholipids				
PC.ae.C40.4	Glycerophospholipids				
PC.ae.C40.5	Glycerophospholipids				
PC.ae.C40.6	Glycerophospholipids				
PC.ae.C42.1	Glycerophospholipids				
PC.ae.C42.2	Glycerophospholipids				
PC.ae.C42.3	Glycerophospholipids				
PC.ae.C44.3	Glycerophospholipids				
PC.ae.C44.5	Glycerophospholipids				
PC.ae.C44.6	Glycerophospholipids				
SM..OH..C14.1	Sphingolipids				
SM..OH..C16.1	Sphingolipids				
SM..OH..C22.1	Sphingolipids				
SM..OH..C22.2	Sphingolipids				
SM..OH..C24.1	Sphingolipids				
SM.C16.0	Sphingolipids				
SM.C16.1	Sphingolipids				
SM.C18.0	Sphingolipids				
SM.C18.1	Sphingolipids				
SM.C20.2	Sphingolipids				
SM.C24.0	Sphingolipids				
SM.C24.1	Sphingolipids				
SM.C26.0	Sphingolipids				
SM.C26.1	Sphingolipids				

**Table 7.** Timing of peak of each final candidate metabolite in the blood for each treatment group. For LF and TRF groups ZT 0/ 0 hours is the start of the feeding window and the time of lights on for LF, while for DF ZT0 is the start of the fasting window and the time of lights on. LF and DF groups are in 12h:12h light:dark although feeding in the day and night, respectively. TRF are in constant darkness although feed for the same 12h window (0–12 hours) as the LF group (same experimenter time). DF=dark fed wild type mice, LF=light fed wild type mice, TRF=time restricted fed Per1/2-null mice.

Metabolite	Class	DF (ZT, hours)	LF (ZT, hours)	TRF (hours)
C14.1	Acylcarnitines	7.12	20.85	19.13
C16	Acylcarnitines	6.83	19.97	20.44
C18.1	Acylcarnitines	6.65	19.92	20.18
Ala	Amino.acid	19.17	7.99	7.38
Asn	Amino.acid	18.47	6.39	6.03
Asp	Amino.acid	17.46	20.42	18.01
Glu	Amino.acid	17.59	20.08	18.38
Ile	Amino.acid	21.03	7.70	0.40
Leu	Amino.acid	20.36	6.65	2.85
Met	Amino.acid	19.90	6.20	5.90
Phe	Amino.acid	19.78	6.99	5.91
Pro	Amino.acid	19.08	6.95	6.34
Thr	Amino.acid	20.06	7.93	6.33
Val	Amino.acid	20.50	7.95	5.05
ADMA	Biogenic_amine	9.74	20.85	19.43
Carnosine	Biogenic_amine	16.39	20.41	19.03
Histamine	Biogenic_amine	15.27	19.06	19.21
Met.SO	Biogenic_amine	19.37	8.04	6.54
Putrescine	Biogenic_amine	15.80	19.05	16.13
SDMA	Biogenic_amine	9.83	20.78	20.54
Serotonin	Biogenic_amine	13.31	6.29	18.74
Spermidine	Biogenic_amine	16.54	19.78	19.13
Taurine	Biogenic_amine	16.07	20.22	19.60
lysoPCaC16:1	Glycerophospholipids	6.50	18.17	18.20
lysoPCaC18:1	Glycerophospholipids	6.97	18.57	16.64
lysoPCaC18:2	Glycerophospholipids	5.90	15.27	12.05
PCaaC32:1	Glycerophospholipids	1.41	16.26	18.89
PCaaC32:2	Glycerophospholipids	23.87	16.15	18.57
PCaaC34:4	Glycerophospholipids	6.71	17.92	0.54
PCaaC38:3	Glycerophospholipids	7.48	19.01	22.51
PCaaC38:4	Glycerophospholipids	8.18	21.07	1.18
PCaaC38:5	Glycerophospholipids	7.70	20.03	0.89
PCaaC38:6	Glycerophospholipids	6.81	20.36	23.97
PCaaC40:4	Glycerophospholipids	5.80	18.63	23.57
PCaaC40:5	Glycerophospholipids	7.01	19.68	23.88
PCaeC34:1	Glycerophospholipids	23.59	16.45	18.99
PCaeC34:3	Glycerophospholipids	0.48	15.22	14.65
PCaeC36:2	Glycerophospholipids	22.36	12.23	10.04
PCaeC38:0	Glycerophospholipids	7.80	19.97	0.71
PCaeC38:2	Glycerophospholipids	20.45	12.78	9.58
PCaeC38:3	Glycerophospholipids	2.18	16.49	19.91
PCaeC42:1	Glycerophospholipids	8.78	20.70	2.72

**Table 8.** Potential for acylcarnitines, glycerophospholipids and amino acids/amines candidates that associate with the feeding-fasting cycle to explain the intraerythrocytic development cycle (IDC) schedule. Requirements for these metabolites is likely to increase as each parasite cell progresses through its IDC.

Metabolite class & general function	Role in the IDC?	Putative time-cue?
**Acylcarnitines** • Energy metabolism • Mitochondrial function • Fatty acid transport	No evidence a lack of acylcarnitines affects either the IDC schedule or replication rate. Peak during fasting when parasites are in their least energetically/ metabolically demanding stages.	Very unlikely.
**Glycerophospholipids** • Cellular signalling and trafficking • Membrane neogenesis • Haemozoin formation	Phospholipid composition of the host RBC increases during infection, especially oleic acid (18:1) ( Déchamps *et al.*, 2010). Parasites scavenge fatty acids and also lysoPCs from host plasma, which compete with each other as a source of the acyl components required for lipid synthesis. Lysophosphatidylcholine a C18:1 (lysoPC a 18:1), which contains an oleic acid side chain, is associated with IDC progression. Majority peak during host fasting when parasites are in their least energetically/ metabolically demanding stages. Thus, to be a time-of day cue	Unlikely. Not essential; unlike the liver stage, IDC stages can synthesise fatty acids de novo via type II FA synthase (FASII), ( Tarun *et al.*, 2009).
**Biogenic amines** **and amino acids:** • Nucleic acid and protein synthesis	Parasites must scavenge several amino acids from the host, including six host-‘essential’ (isoleucine, leucine, methionine, phenylalanine, threonine and valine) and one host-‘non-essential’ (alanine) amino acid ( Payne & Loomis, 2006) that are rhythmic. Parasites can scavenge most amino acids from catabolism of host haemoglobin ( Babbitt *et al.*, 2012; Liu *et al.*, 2006; Martin & Kirk, 2007). Isoleucine is the only amino acid absent from human haemoglobin and is one of the least abundant amino acids in rodent haemoglobin (1-3%, Figure 9) yet makes up 9% of both *P. falciparum’s* and *P. chabaudi’s* amino acids ( Yadav & Swati, 2012). The response of *P. falciparum* to isoleucine withdrawal is not influenced by whether they were previously cultured in a high or low isoleucine concentration environment and results in dormancy that can be broken upon isoleucine replenishment ( Babbitt *et al.*, 2012). Coinciding with the rise in isoleucine concentration during the feeding window, trophozoites transition to schizonts before bursting and beginning development as ring stages at the end of the feeding window ( Figure 6, Figure 8).	Likely. Isoleucine is the only essential amino acid for *P. falciparum*, it cannot be stored, and dormancy has no apparent ill-effects on subsequent development. *P. falciparum* deprived of other amino acids develop as normal ( Liu *et al.*, 2006).

### Timing and completion of the parasite IDC depends on the availability of isoleucine

That malaria parasites rely on exogenous isoleucine as both a resource and time-cue to complete the IDC is supported by previous observations across species of *Plasmodium*. First, when the human malaria *P. falciparum* is deprived of isoleucine, parasites immediately and dramatically slow cell cycle progression akin to dormancy ( Liu *et al.*, 2006) yet are able to recover even after long periods of starvation (~4 IDCs) ( Babbitt *et al.*, 2012). Second, in contrast to isoleucine, if other amino acids are removed from culture media, parasites switch to scavenging them from haemoglobin with only minor effects on IDC completion ( Babbitt *et al.*, 2012). Third, not only is isoleucine crucial for the development of *P. knowlesi* in culture, parasites only incorporate isoleucine for part of the IDC (until the point that schizogony starts ( Butcher & Cohen, 1971; Polet & Conrad, 1968; Polet & Conrad, 1969; Sherman, 1979)). Fourth, isoleucine is one of few amino acids that exhibits a daily rhythm in the blood of mice and humans. Specifically, isoleucine was inverted with a 12-hour shift in simulated day and night shift working humans (a phase difference of 11:49 ± 02:10 h between the day and night shift conditions) and follows the timing of food intake ( Skene *et al.*, 2018). Therefore, our next experiments tested whether an exogenous supply of isoleucine is capable of scheduling the IDC.

**Figure 7.**
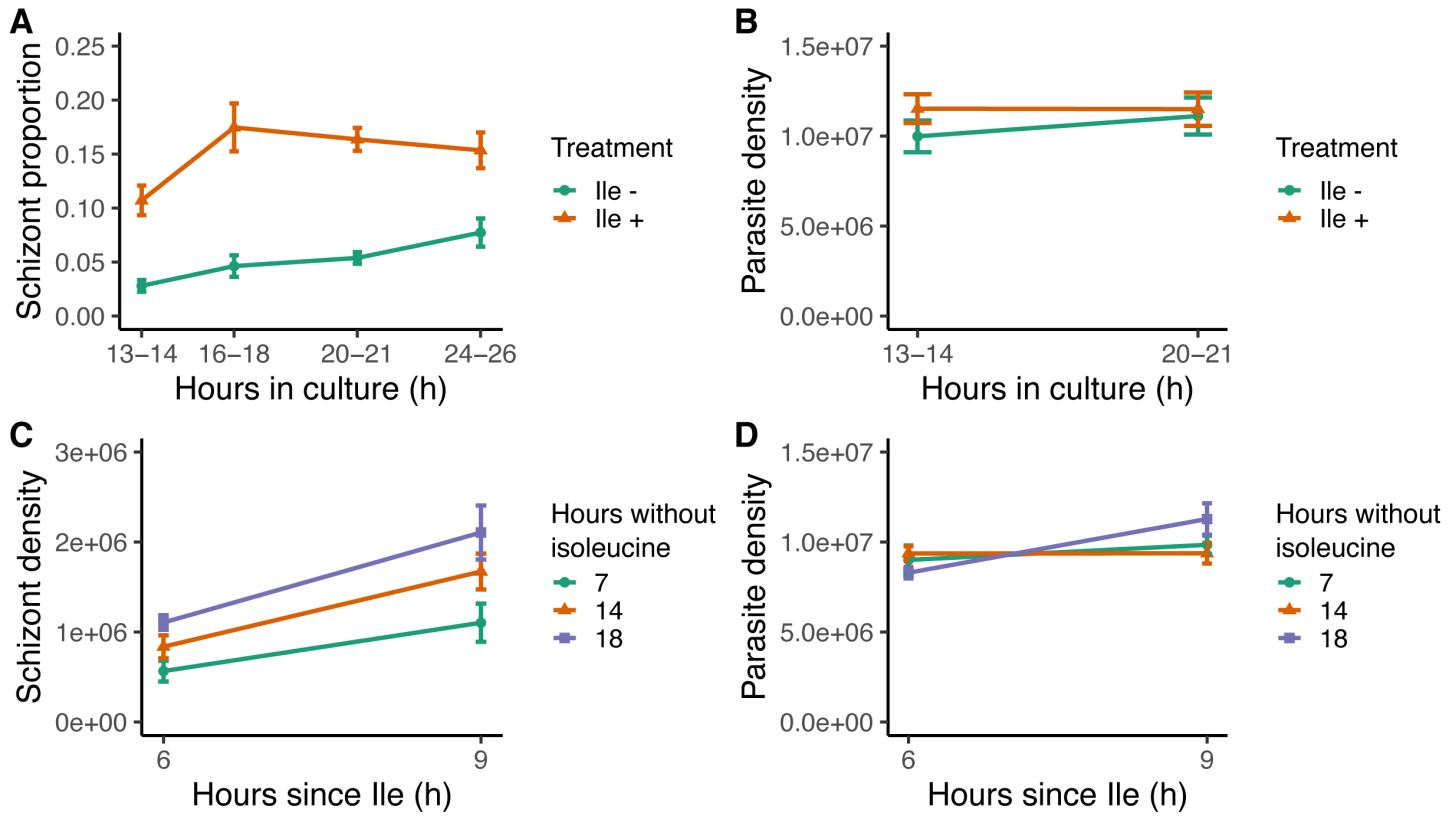
Isoleucine provision and withdrawal drives intraerythrocytic development cycle (IDC) completion but does affect parasite mortality. ( **A**) IDC completion defined as the proportion of parasites that are schizonts, in cultures with (orange triangles, Ile +, 50 mg/L) or without (green circles, Ile -) isoleucine. ( **B**) Density of all parasite stages when parasites are cultured with or without isoleucine. Density of ( **C**) schizonts and ( **D**) all parasite stages, after the addition of isoleucine into cultures following isoleucine deprivation for seven (green circles), 14 (orange triangles) and 18 hours (purple squares). The proportion of schizonts in the blood seeding the cultures was ~0.005.

We carried out two experiments in parallel to quantify how *P. chabaudi’s* IDC progression is affected when deprived of isoleucine, and whether the IDC is then completed (defined as the proportion of parasites that reach the schizont stage) when isoleucine is restored. We conducted these experiments *in vitro* where isoleucine levels and timing can be controlled more successfully than by attempting to perturb blood isoleucine concentration *in vivo.* The challenges of an *in vivo* manipulation include that isoleucine is essential for mice so hosts cannot be maintained in isoleucine-free conditions without negative health consequences, particularly via its roles in the immune system and response to anaemia ( Mao *et al*., 2018; Murata & Moriyama, 2007; Rivas-Santiago *et al*., 2011). These potential off-target effects could confound conclusions about how the IDC behaves *in vivo*. First, parasites cultured in the absence of isoleucine (n=32 cultures from the blood of eight mice, which were split equally across both treatments) develop extremely slowly with approximately three-fold fewer completing the IDC compared to parasites with isoleucine (50 mg/L, which is the same concentration as standard culture media; RPMI 1640) (n=32 cultures) ( Figure 7A). The best fitting model contained only “treatment” (parasites cultured with or without isoleucine) as a main effect (ΔAICc=0, Table 9A). The reduction in schizonts in isoleucine-free conditions was not due to a higher death rate because the density of parasites remains constant during the experiment and did not differ between the treatments ( Figure 7B) (best fitting model is the null model ΔAICc=0, Table 9B). Further, incorporating either “treatment” or “hours in culture” into the model did not improve the model fit (treatment: ΔAICc=4.16, hours in culture: ΔAICc=5.50, Table 9B). This experiment extends previous findings for *P. falciparum* ( Babbitt *et al.*, 2012; Liu *et al.*, 2006; McLean & Jacobs-Lorena, 2020) across species as well as provides greater resolution on the parasite phenotypes involved. 

**Table 9.** Degrees of Freedom (df), log-Likelihood (logLik), AICc, ΔAICc (AICc _i_ – AICc _min_) and AICc w (AICc weight) for each linear model in the schizont/parasite proportion/density analysis ordered in descending fit (best-fitting model at the top). The response variable for each model is either schizont proportion (Schizont.prop), parasite density (Parasite.dens) or schizont density (Schizont.dens) and the random effect is “mouseID”. “Treatment” refers to the treatment group (DF, LF, TRF or ALF), “hours in culture” refers to the number of hours spent in culture since being extracted from the mice, and “hours since isoleucine” refers to the number of hours since isoleucine was added to the cultures. Treatment, hours in culture and hours since isoleucine were all fitted as factors. DF=dark fed wild type mice, LF=light fed wild type mice, TRF=time restricted fed Per1/2-null mice, ALF=ad libitum fed Per1/2-null mice.

Model description: A) Schizont.prop ~ + (1|mouseID)	df	logLik	AICc	ΔAICc	AICc *w*
treatment	4	106.26	-203.8	0.00	0.996
treatment + hours in culture	7	104.29	-192.6	11.26	0.004
treatment * hours in culture	10	98.81	-173.5	30.37	0.000
null	3	80.96	-155.5	48.31	0.000
hours in culture	6	74.89	-136.3	67.54	0.000
B) Parasite.dens ~ + (1|mouseID)					
null	3	0.14	6.6	0.00	0.836
treatment	4	-0.63	10.7	4.16	0.104
hours in culture	4	-1.30	12.1	5.50	0.053
treatment + hours in culture	5	-2.07	16.4	9.88	0.006
treatment * hours in culture	6	-2.79	20.9	14.36	0.001
C) Schizont.dens ~ + (1|mouseID)					
treatment + hours since isoleucine	6	69.67	-125.3	0.00	0.887
hours since isoleucine	4	65.04	-121.2	4.14	0.112
treatment * hours since isoleucine	8	65.67	-111.6	13.65	0.001
null	3	56.87	-107.2	18.10	0.000
treatment	5	56.67	-101.9	23.39	0.000
D) Parasite.dens ~ + (1|mouseID)					
hours since isoleucine	4	12.96	-17.0	0.00	0.568
null	3	11.49	-16.4	0.56	0.430
treatment + hours since isoleucine	6	9.10	-4.1	12.85	0.001
treatment	5	7.76	-4.1	12.90	0.001
treatment * hours since isoleucine	8	10.47	-1.2	15.75	0.000

**Figure 8.**
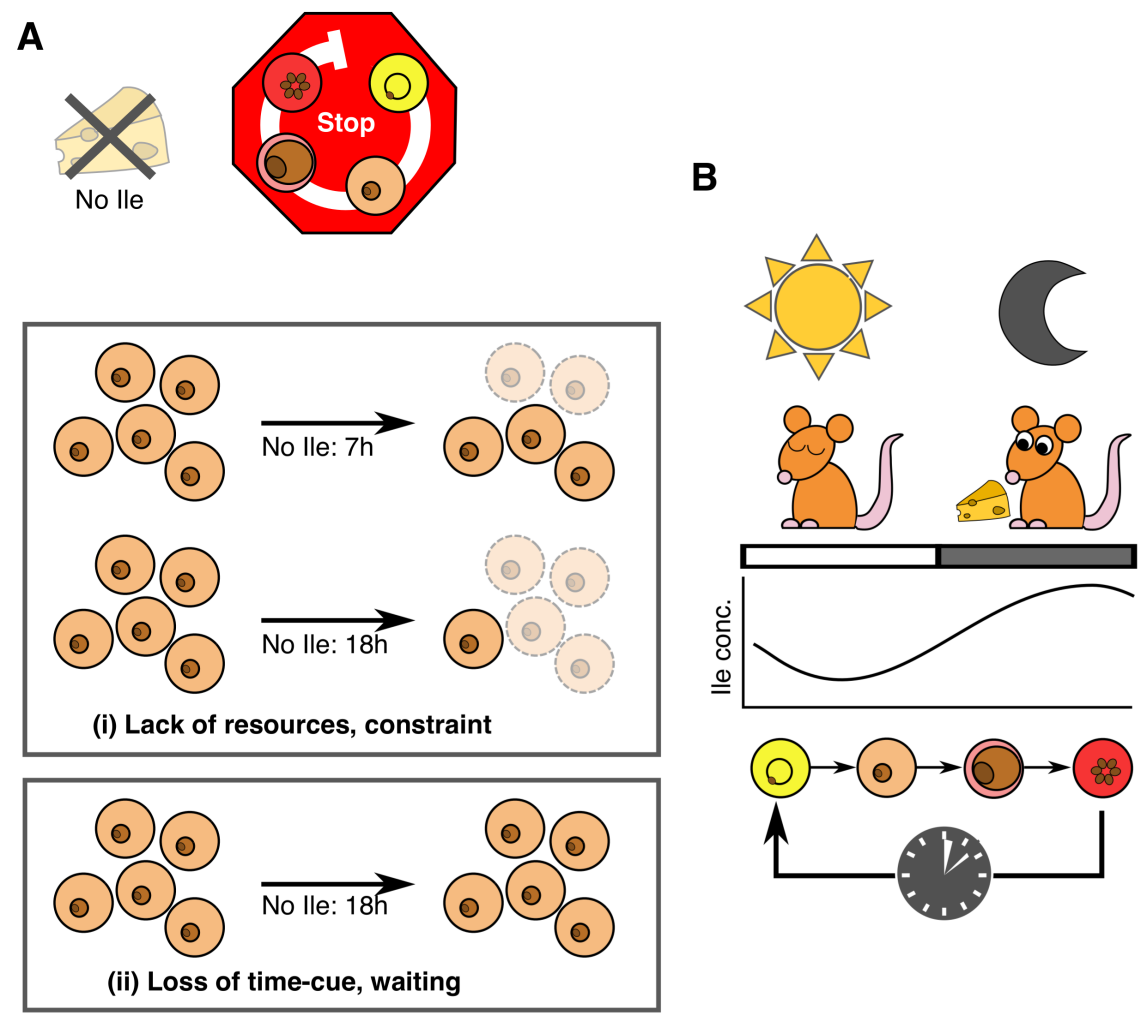
Schematic for isoleucine’s role in the intraerythrocytic development cycle (IDC) schedule. ( **A**) In the absence of isoleucine (Ile), but in the presence of all other essential components of culture media, *P. chabaudi’s* IDC progression stops or continues very slowly, as observed for *P. falciparum* ( Liu *et al.*, 2006 and Babbitt *et al.*, 2012). This observation is consistent with isoleucine being an essential resource and/or a time-cue. This means the time-of-day cue may also be a direct selective agent (i.e. the reason why a timing strategy has evolved). In such situations it is very difficult to unravel the consequences of cue loss. Here, the challenge is differentiating to what extent the absence of isoleucine (i) creates a non-permissive environment, imposing a constraint on parasites that simply forces IDC progression to stop, versus (ii) parasites responding in a strategic manner and ‘deciding’ to stop development. Under scenario (i) parasites experience resource limitation, and starvation by definition should have negative consequences. For example, the longer parasites are starved, the more die, or experience increasingly lower or slower rates of recovery upon isoleucine replenishment. Under scenario (ii) there are no such costs because parasites avoid starvation by stopping/slowing development and waiting for a signal that it is time to expect resources to be replenished. That parasite number is not affected by isoleucine withdrawal, even for prolonged periods, and that parasites recover IDC completion at the same rate regardless of the duration of withdrawal, is consistent with scenario (ii) not scenario (i). ( **B**) It is now well established that *P. chabaudi’s* IDC schedule is synchronised to host feeding-fasting cycles. Our findings suggest this coordination is achieved by parasites responding to daily rhythmicity in the blood concentration of isoleucine for the purpose of maximising exploitation of the host’s isoleucine, and potentially other nutrients, they can more easily acquire from the host’s digestion of its food than by biosynthesis or scavenging from haemoglobin.

The substantial slowing of IDC progression in the absence of isoleucine is further supported by our second experiment. This experiment revealed that like for *P. falciparum, P. chabaudi* completes IDC development when isoleucine deprivation ends. Parasites (~10 ^7^ per culture) were added to isoleucine-free media and incubated for seven, 14, or 18 hours, after which isoleucine (50 mg/L) was added to their cultures (n=16 cultures per treatment). Parasites completed development when isoleucine became available, regardless of the duration of deprivation (seven, 14, or 18 hours), with the best fitting model containing main effects of “treatment” and “hours since isoleucine added” (ΔAICc=0, Table 9C). Importantly, including the interaction did not improve the model fit (ΔAICc=13.65, Table 9C), demonstrating that IDC completion proceeds at the same rate despite different durations of isoleucine starvation. Specifically, the rate of IDC completion in the 6–9 hours following isoleucine replenishment was approximately 50% for all groups ( Figure 7C). Again, higher death rates in cultures deprived of isoleucine for the longest time period did not give the appearance of equal IDC rates because the model incorporating “hours since isoleucine” was competitive with the null model (ΔAICc=0.56, Figure 7D, Table 9D), revealing parasites were still as viable after 18 hours of deprivation as those deprived for seven and 14 hours. Furthermore, cultures deprived the longest achieved the most schizonts (18 hours, mean±SEM: 1.61×10 ^6 ^±0.20 Figure 7C), while the fewest schizonts were observed in cultures deprived for the shortest period (seven hours, mean±SEM: 0.83×10 ^6 ^±0.14 Figure 7C). Unlike previous studies, by comparing rates of completion across treatments (i.e. statistically testing the roles of the duration of deprivation and its interaction with treatment), our experiment demonstrates that isoleucine deprivation is not costly to parasites ( Figure 8A). The variation in the intercepts of Figure 7C (<1% schizonts after seven hours, 3% after 14 hours and 5% after 18 hours deprivation) is likely explained by the 18 hour deprivation cultures accumulating a higher proportion of schizonts at the time of isoleucine provision simply as a product of developing very slowly during a longer window of deprivation (in line with Liu *et al.*, 2006 and Babbitt *et al.*, 2012). The lack of costs and ability to recover development when isoleucine is replenished - which differs from the response to the loss of other essential nutrients - is consistent with isoleucine being sufficient to act as a time-cue as well as being an essential resource ( Figure 8B).

## Discussion

Our large-scale metabolomics screening experiment ( Figure 3, Figure 4, Figure 6, Table 8) and follow-up experiments ( Figure 5, Figure 7), demonstrate that isoleucine is sufficient to explain how malaria parasites schedule their IDC in line with host feeding-fasting rhythms. A key challenge was differentiating to what extent the absence of isoleucine creates a non-permissive environment, imposing a constraint on parasites that simply forces IDC progression to stop, versus parasites responding in a strategic manner and ‘deciding’ to stop development ( Figure 8A). There are several reasons why our findings are not a case of revealing the obvious, that parasites cannot develop when resource-limited. First, when deprived of other essential resources such as glucose, *P. falciparum* rapidly displays stress responses at the transcriptional level yet when deprived of isoleucine, its normal transcriptional pattern persists, albeit very slowly ( Babbitt *et al.*, 2012), suggesting it is waiting for a signal to proceed (a “gate”). Second, parasites cannot cope with deprivation of other essential resources, for example dying within hours of glucose deprivation ( Babbitt *et al.*, 2012), yet the IDC restarts with no apparent ill-effects when isoleucine is replenished ( Figure 7). Third, that parasites cannot create isoleucine stores, nor generate it endogenously, and mount rapid transcriptional responses to changes in exogenous isoleucine concentration ( Babbitt *et al.*, 2012), are all hallmarks of an informative cue and a sensitive response system. Thus, we propose that either parasites are so well adapted to isoleucine starvation they cope just fine without it, unlike their ability to cope with other forms of starvation, or that isoleucine exerts its effects on IDC progression as both a time-cue and resource ( Figure 8B).

Most studies of isoleucine uptake and use in malaria parasites focus on *P. falciparum*. This parasite uses several channels and receptors (both parasite- and host-derived) to acquire resources from the host. Uninfected human RBC take up both isoleucine and methionine via the saturable L-system ( Cobbold *et al.*, 2011), which supplies 20% of the necessary isoleucine ( Martin & Kirk, 2007). When parasitised, there is a five-fold increase in isoleucine entering RBC which is attributable to new permeability pathways (NPPs) introduced into the RBC membrane by the parasite ( Martin & Kirk, 2007; McLean & Jacobs-Lorena, 2020). NPPs supply 80% of the necessary isoleucine ( Martin & Kirk, 2007) and are active only in the host membrane at the trophozoite and schizont stages of the IDC, suggesting this influx of isoleucine occurs only at certain times of day (e.g. after host feeding) ( Kutner *et al.*, 1985). Assuming *P. chabaudi* has analogous mechanisms, we propose that elevated isoleucine is used by the parasite as a marker for a sufficiently nutrient-rich environment to traverse cell cycle checkpoints and complete the IDC ( McLean & Jacobs-Lorena, 2020; O’Neill *et al.*, 2020).

Glucose has previously been suggested as a time-cue or scheduling force for the IDC ( Hirako *et al.*, 2018; Prior *et al.*, 2018). Our experiment - which is the first to quantify glucose and IDC rhythms across different feeding-fasting rhythms and expose parasites to artificial glucose rhythms in arrhythmic hosts - does not support a direct effect of glucose but it may be indirectly involved. Parasites that are glucose-limited fail to concentrate isoleucine ( Martin & Kirk, 2007), likely due to a lack of glycolysis and ATP production needed to operate isoleucine transporters. High concentrations of isoleucine in the blood are also associated with uptake of glucose by tissues, potentially contributing to the hypoglycaemia associated with TNF-stimulation of immune cells during malaria infection ( Elased & Playfair, 1994; Hirako *et al.*, 2018). Additionally, rodent models and humans with obesity and type 2 diabetes-like pathologies have elevated levels of isoleucine and dampened glucose rhythms in the blood ( Lynch & Adams, 2014; Isherwood *et al.*, 2017). Thus, if glucose limitation or elevation interferes with the parasite’s ability to acquire time-of-day information from isoleucine, the IDC schedule will be disrupted, as observed in diabetic mice ( Hirako *et al.*, 2018). Connections between isoleucine and glucose might also explain why the parasite protein kinase ‘KIN’ is involved in nutrient sensing ( Mancio-Silva *et al.*, 2017). Whilst isoleucine appears to be the main driver of the IDC schedule, glucose likely mediates isoleucine’s impact via multiple, non-mutually exclusive, activities.

**Figure 9.**
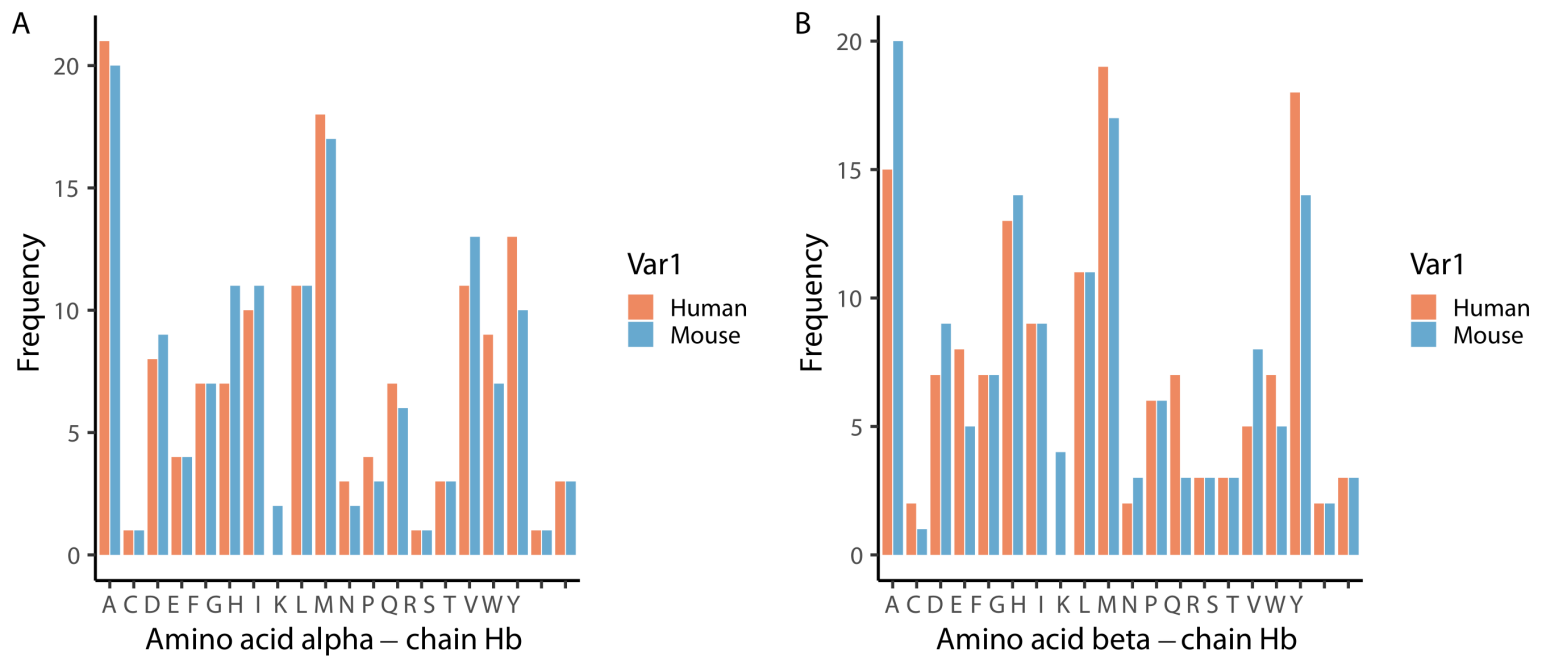
Frequency of amino acids in alpha ( **A**) and beta ( **B**) chains of human (orange) and mouse (blue) haemoglobin. Amino acid codes: A - alanine, C - cysteine, D - aspartic acid, E - glutamic acid, F - phenylalanine, G - glycine, H - histidine, I - isoleucine, K - lysine, L - leucine, M - methionine, N - asparagine, P - proline, Q - glutamine, R - arginine, S - serine, T - threonine, V - valine, W - tryptophan, Y - tyrosine.

Given that daily variation in the concentration of isoleucine in the blood of infected mice appears modest (55 μM to 80 μM from nadir to peak; Figure 6), we suggest that like *P. falciparum, P. chabaudi* is very sensitive to changes in isoleucine levels. This observation could be interpreted in two non-mutually exclusive ways. Perhaps this seemingly damp rhythm means that sufficient isoleucine is available around-the-clock to meet the parasites resource-needs (from the blood and scavenging from the very low level (<3%) of isoleucine in rodent haemoglobin; Figure 9), so isoleucine acts on the IDC schedule only as a time-cue (to align with resources that are limited at certain times of day) rather than as a developmental-rate limiting resource as well. For instance, the appearance of isoleucine may signal a window during which vitamins, cofactors, purines, folic acid, pantothenic acid, and glucose, that are also required for successful replication, are available ( Müller & Kappes, 2007; Müller *et al.*, 2010; Sherman, 1979). Furthermore, isoleucine is also a reliable cue for the timing of other nutrients that are perhaps easier for parasites to acquire from the host’s digestion of food than from biosynthesis or scavenging from haemoglobin. In particular, that the expression of genes associated with translation are the most commonly disrupted when *P. chabaudi’s* rhythms are perturbed away from the host’s rhythms ( Subudhi *et al.*, 2020) suggests parasites align the IDC schedule with the resources required for proteins. However, if isoleucine is not a limiting resource at certain times of day, this may only be the case early infections.

Like blood glucose concentration, the isoleucine rhythm may become exacerbated as infections develop high parasite burdens and hosts become sick. In this case, by aligning the IDC schedule correctly early in infections, parasites minimise the costs of resource limitation later on.

Our findings offer a route into identifying the molecular pathways involved in transducing environmental signals to the IDC schedule. To date, the only gene known to be involved in the IDC schedule is SR10, which modulates IDC duration in response to perturbations of host time-of-day ( Subudhi *et al.*, 2020). Identifying KIN ( Mancio-Silva *et al.*, 2017) regulated pathways and whether they, along with pathways associated with SR10 ( Subudhi *et al.*, 2020), and PK7/MORC (which is involved in IDC stage transitions; Singh *et al.*, 2021), are sensitive to isoleucine might reveal how the parasites’ time-keeping mechanism operates. Whilst by no means conclusive, our results feed the debate about whether malaria parasites keep time by an endogenous circadian oscillator or a simpler mechanism. The hallmarks of an endogenous circadian clock are (i) temperature compensation, (ii) free running in constant conditions, and (iii) entrainment to a Zeitgeber ( Pittendrigh, 1960). Recent observations are consistent with (ii) ( Rijo-Ferreira *et al.*, 2020; Smith *et al.*, 2020; Subudhi *et al.*, 2020) and our results now allow entrainment to isoleucine to be tested for (iii), as well as free-running in isoleucine-constant conditions (ii). However, an endogenous clock may exist to allow parasites to schedule activities other than the IDC. Overall, the rapid stop-start responses to isoleucine withdrawal and replenishment that we observe, and that is known for *P. falciparum* ( Babbitt *et al.*, 2012; Liu *et al.*, 2006; McLean & Jacobs-Lorena, 2020) *,* appear more parsimoniously explained by a “just-in-time reactionary strategy” than an endogenous oscillator. This is because clocks generally require several cycles to catch up with a large change in the phase (timing) of their Zeitgeber (which is why jet lag occurs). Thus, in the absence of isoleucine or a phase-shift in its availability, if the ticking of an endogenous clock cannot be readily de-coupled from IDC progression, development would have continued as best as possible, instead of stopping. Furthermore, most clocks are set by a Zeitgeber (usually light) that is a good proxy for something else (e.g. predation risk) that is the ultimate driver of selection for a timing mechanism. Yet, isoleucine appears to be both cue and selective driver, further suggesting a just-in-time response. This strategy has the advantage of cueing in to the most accurate information about resource availability, and providing a more fast-responding strategy than a clock when the bulk of host foraging could occur anytime within the broad window of the active phase, and whose timing may be disrupted if hosts become severely ill.

By whatever mechanism the IDC is scheduled, coordinating development according to the availability of the resources needed to produce progeny intuitively seems like a good strategy to maximise fitness. Yet, the costs/benefits of the IDC schedule demonstrated by parasites may be mediated by parasite density. At low parasite densities (e.g. at the start of infection), resources may be sufficient to support IDC completion of all parasites at any time-of-day, but at intermediate densities, parasites may need to align their IDC needs with rhythms in resource availability. Finally, at very high densities and/or when hosts become sick, resources could be limiting so a synchronous IDC leads to deleterious competition between related parasites. Quantifying whether the costs and benefits of a synchronous IDC vary during infections in line with parasite density and resource availability is complicated by the connection between the IDC schedule and the timing of transmission stages. By aligning the IDC schedule with the feeding-fasting rhythm which is set by light-dark rhythms in the environment, parasites benefit from the knock-on alignment of transmission stage maturation with vector biting activity ( O’Donnell *et al.*, 2011; Pigeault *et al.*, 2018; Schneider *et al.*, 2018). Therefore, a key question is to what extent does the IDC schedule contribute to parasite fitness via transmission benefits versus variation during infections in the benefits and costs of aligning asexual replication with rhythmic resources in the blood? More broadly, by integrating evolutionary and mechanistic insight, it may be possible to improve antimalarial treatments. For example, impairing the parasites ability to detect or respond to isoleucine may stall the IDC, reducing virulence and buy time for host immune responses to clear the infection, as well as preventing ring stage dormancy from providing tolerance to antimalarials ( Codd *et al.*, 2011).

## Methods

### Ethics statement

All procedures complied with the UK Home Office regulations (Animals Scientific Procedures Act 1986; project 483 licence number 70/8546) and were approved by the University of Edinburgh. All efforts were made to ameliorate harm to animals through monitoring of animal health by twice daily visual inspections and daily routine health checks, such as weight and RBC count.

### Experimental designs and terminology

The same four perturbations of host and parasite rhythms were used in the metabolomics experiment and the first glucose monitoring experiment. The Per1/2-null mice (non-functional proteins Period 1 & 2, backcrossed onto a C57BL/6 background for over 10 generations) were donated by Michael Hastings (MRC Laboratory of Molecular Biology, Cambridge, UK) and generated by David Weaver (UMass Medical School, Massachusetts, USA). Wild type C57BL/6 mice were housed in a 12h light: 12h dark regime (12 hours of light followed by 12 hours of darkness) and the Per1/2-null mice housed in constant darkness (DD). All mice acclimated to their photo schedule and feeding treatments for two weeks prior to the start of infections and conditions were maintained throughout sampling. We randomly separated mice into their treatment groups. We refer to time-of-day using Zeitgeber Time (ZT) for mice housed under entrained conditions (light:dark cycles) and hours when Per1/2-null mice are housed under constant conditions (dark:dark). WT mice either had access to food at night (dark feeding, DF) or in the day (light feeding, LF). Per1/2-null mice either had access to food for 12 hours (time restricted feeding, TRF) or constant access to food ( *ad libitum* feeding, ALF). Every 12 hours, food was added/removed accordingly from the DF, LF and TRF cages and the cages were checked for evidence of hoarding, which was never observed. ALF cages were also disturbed during food removal/provision of the other groups. Investigators could not be blinded to the mouse strain due the difference in coat colour, or the lighting and feeding regime due to the need to add and remove food manually.

***Sampling*.** All mice were infected intravenously with *P. chabaudi* DK genotype by injection of 1×10 ^5^ infected RBC at ring stage. Sampling started on day 5 post infection and occurred every two hours for both the metabolomics and glucose experiments. We made a thin blood smear each time a mouse was sampled to quantify parasite rhythms by counting ~100 parasites per blood smear using microscopy. Smears were read without knowledge of the treatment group, and excluded where smear quality was too poor to count RBCs. Following Prior *et al.* (2018); O’Donnell *et al.* (2020) and Rijo-Ferreira *et al.* (2020), we used the proportion of ring stages as a phase marker (an estimate of the timing of parasite development in the blood) of parasite rhythms. We calculated amplitude and time-of-day of peak for each treatment group using sine and cosine curves in a linear model to confirm the IDC schedules for each group as used in O’Donnell *et al.* (2020).

***Metabolomics experiment*.** We infected 68 eight-week-old female mice: 35 C57BL/6 wild type animals (DF and LF mice) and 33 Per1/2-null TTFL clock-disrupted mice (TRF and ALF) ( Table 2). Sample sizes were chosen based on previous data collected from our lab using our study system. We sampled mice in blocks (A–D), meaning each individual mouse was sampled every eight hours during the 26-hour sampling window, with 14 time points in total. We did not sample each mouse at each sampling point to minimise the total volume of blood being taken. At each sampling point for each designated host, 20 µl blood was taken from the tail vein to provide 10 µl blood plasma for snap freezing using dry ice.

***Glucose monitoring*.** We infected 20 eight-week-old male mice: 10 C57BL/6 wild type animals and 10 Per1/2-null circadian clock-disrupted mice (as described above, Table 2). Sample sizes were chosen based on previous data collected using our study system. We recorded blood glucose concentration from all mice every two hours by taking 1 µl blood using an Accu-Chek Performa Nano glucometer.

***Glucose perturbations.*** We infected 16 eight-to-ten week old male Per1/2/-null mice (housed in DD to ensure arrhythmicity) with 1×10 ^5^ infected RBC at ring stage (note, a 10 fold higher density than all the other experiments). From day 1 post infection, intra-peritoneal injections of 100µl glucose (3g/kg of glucose, n=5), glucose + insulin (4.5IU/kg of insulin + 3g/kg of glucose, n=6) or PBS (n=5) occurred every 24h up to and including day 6 post infection. Injections occurred at the same time every day, at 12 hours out of phase with the IDC schedule of the parasites at the point of infection (i.e. 12 hours out of phase with peak feeding in the donor mice). Mice were sampled on days 5-6 at 0h, injections administered at 1h, then sampled again at 2h and 4h after which all mice were sampled every 4 hours up to and including 24h after the first sample. Samples consisted of a thin blood smear to stage parasites as well as calculate parasite density, blood glucose concentration ( Accu-Chek Performa Nano glucometer), and RBC density (Beckman Coulter).

### Targeted metabolomics

**Figure 10.**
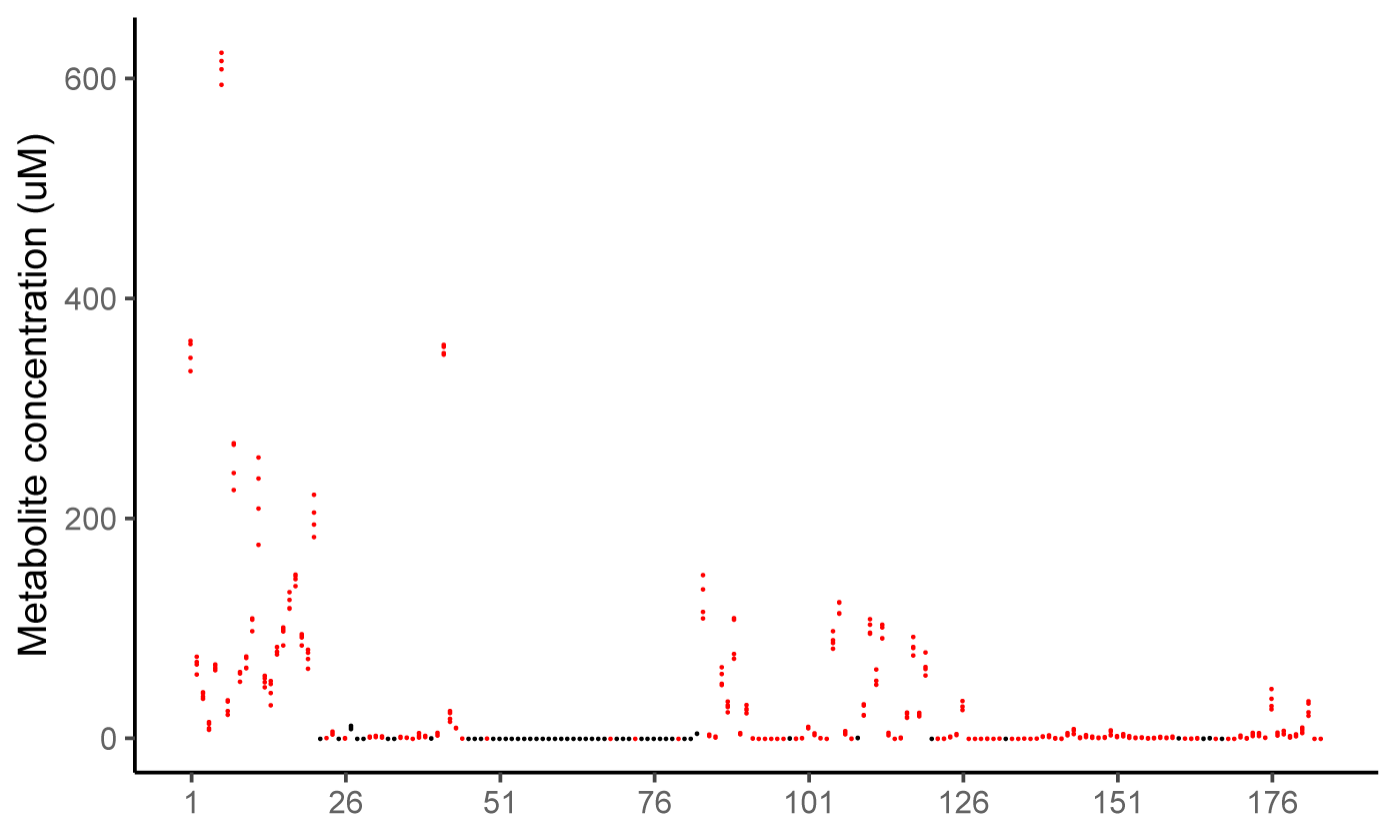
Median concentration of all metabolites across the time series for all groups, those included and excluded from the analysis. The median concentration (µM) of each metabolite for each of the 4 treatment groups. In black are metabolites excluded since they failed set LC/MS assay criteria and those metabolites taken forward into the analysis are red. The numbers on the x axis correspond to the metabolites as follows: (1)Ala, (2)Arg, (3)Asn, (4)Asp, (5)Cit, (6)Gln, (7)Glu, (8)Gly, (9)His, (10)Ile, (11)Leu, (12)Lys, (13)Met, (14)Orn, (15)Phe, (16)Pro, (17)Ser, (18)Thr, (19)Trp, (20)Tyr, (21)Val, (22)Ac.Orn, (23)ADMA, (24)alpha.AAA, (25)c4.OH.Pro, (26)Carnosine, (27)Creatinine, (28)DOPA, (29)Dopamine, (30)Histamine, (31)Kynurenine, (32)Met.SO, (33)Nitro.Tyr, (34)PEA, (35)Putrescine, (36)Sarcosine, (37)SDMA, (38)Serotonin, (39)Spermidine, (40)Spermine, (41)t4.OH.Pro, (42)Taurine, (43)C0, (44)C2, (45)C3, (46)C3.DC..C4.OH., (47)C3.OH, (48)C3.1, (49)C4, (50)C4.1, (51)C5, (52)C5.DC..C6.OH., (53)C5.M.DC, (54)C5.OH..C3.DC.M., (55)C5.1, (56)C5.1.DC, (57)C6..C4.1.DC., (58)C6.1, (59)C7.DC, (60)C8, (61)C9, (62)C10, (63)C10.1, (64)C10.2, (65)C12, (66)C12.DC, (67)C12.1, (68)C14, (69)C14.1, (70)C14.1.OH, (71)C14.2, (72)C14.2.OH, (73)C16, (74)C16.OH, (75)C16.1, (76)C16.1.OH, (77)C16.2, (78)C16.2.OH, (79)C18, (80)C18.1, (81)C18.1.OH, (82)C18.2, (83)lysoPC.a.C14.0, (84)lysoPC.a.C16.0, (85)lysoPC.a.C16.1, (86)lysoPC.a.C17.0, (87)lysoPC.a.C18.0, (88)lysoPC.a.C18.1, (89)lysoPC.a.C18.2, (90)lysoPC.a.C20.3, (91)lysoPC.a.C20.4, (92)lysoPC.a.C24.0, (93)lysoPC.a.C26.0, (94)lysoPC.a.C26.1, (95)lysoPC.a.C28.0, (96)lysoPC.a.C28.1, (97)PC.aa.C24.0, (98)PC.aa.C26.0, (99)PC.aa.C28.1, (100)PC.aa.C30.0, (101)PC.aa.C32.0, (102)PC.aa.C32.1, (103)PC.aa.C32.2, (104)PC.aa.C32.3, (105)PC.aa.C34.1, (106)PC.aa.C34.2, (107)PC.aa.C34.3, (108)PC.aa.C34.4, (109)PC.aa.C36.0, (110)PC.aa.C36.1, (111)PC.aa.C36.2, (112)PC.aa.C36.3, (113)PC.aa.C36.4, (114)PC.aa.C36.5, (115)PC.aa.C36.6, (116)PC.aa.C38.0, (117)PC.aa.C38.3, (118)PC.aa.C38.4, (119)PC.aa.C38.5, (120)PC.aa.C38.6, (121)PC.aa.C40.1, (122)PC.aa.C40.2, (123)PC.aa.C40.3, (124)PC.aa.C40.4, (125)PC.aa.C40.5, (126)PC.aa.C40.6, (127)PC.aa.C42.0, (128)PC.aa.C42.1, (129)PC.aa.C42.2, (130)PC.aa.C42.4, (131)PC.aa.C42.5, (132)PC.aa.C42.6, (133)PC.ae.C30.0, (134)PC.ae.C30.1, (135)PC.ae.C30.2, (136)PC.ae.C32.1, (137)PC.ae.C32.2, (138)PC.ae.C34.0, (139)PC.ae.C34.1, (140)PC.ae.C34.2, (141)PC.ae.C34.3, (142)PC.ae.C36.0, (143)PC.ae.C36.1, (144)PC.ae.C36.2, (145)PC.ae.C36.3, (146)PC.ae.C36.4, (147)PC.ae.C36.5, (148)PC.ae.C38.0, (149)PC.ae.C38.1, (150)PC.ae.C38.2, (151)PC.ae.C38.3, (152)PC.ae.C38.4, (153)PC.ae.C38.5, (154)PC.ae.C38.6, (155)PC.ae.C40.1, (156)PC.ae.C40.2, (157)PC.ae.C40.3, (158)PC.ae.C40.4, (159)PC.ae.C40.5, (160)PC.ae.C40.6, (161)PC.ae.C42.0, (162)PC.ae.C42.1, (163)PC.ae.C42.2, (164)PC.ae.C42.3, (165)PC.ae.C42.4, (166)PC.ae.C42.5, (167)PC.ae.C44.3, (168)PC.ae.C44.4, (169)PC.ae.C44.5, (170)PC.ae.C44.6, (171)SM..OH..C14.1, (172)SM..OH..C16.1, (173)SM..OH..C22.1, (174)SM..OH..C22.2, (175)SM..OH..C24.1, (176)SM.C16.0, (177)SM.C16.1, (178)SM.C18.0, (179)SM.C18.1, (180)SM.C20.2, (181)SM.C24.0, (182)SM.C24.1, (183)SM.C26.0, (184)SM.C26.1.

We quantified metabolites by analysing plasma samples using the AbsoluteIDQ p180 targeted metabolomics kit (Biocrates Life Sciences AG, Innsbruck, Austria) and a Waters Xevo TQ-S mass spectrometer coupled to an Acquity UPLC system (Waters Corporation, Milford, MA, USA) ( Isherwood *et al.*, 2017; Skene *et al.*, 2018). We prepared the plasma samples (10 µl) according to the manufacturer’s instructions, adding several stable isotope–labelled standards to the samples prior to the derivatization and extraction steps. Using UPLC/MS (ultra performance liquid chromatography/mass spectrometry), we quantified 185 metabolites from five different compound classes (acylcarnitines, amino acids, biogenic amines, glycerophospholipids, and sphingolipids). We ran the samples on two 96-well plates, randomised the sample order and ran three levels of quality control (QC) on each plate. We normalised the data between the plates using the results of quality control level 2 (QC2) repeats across the plate (n=4) and between plates (n=2) using Biocrates METIDQ software (QC2 correction). Metabolites were excluded if the CV% of QC2 was >30% or if all four groups contained >25% of samples that were below the limit of detection, below the lower limit of quantification, or above the limit of quantification or blank out of range. The remaining 134 quantified metabolites comprised of seven acylcarnitines, 19 amino acids, 15 biogenic amines, 79 glycerophospholipids and 14 sphingolipids (see Figure 10).

### Isoleucine response experiments

To test the effect of the amino acid isoleucine on the IDC, we compared parasite developmental progression in cultures with and without isoleucine (50 mg/L), as well as after different durations (seven, 14, 18 hours) of isoleucine starvation. We set up N = 112 cultures (from eight mice) so that for each time point within each treatment, an independent culture was sampled, avoiding any bias associated with repeat-sampling individual cultures. We used eight-week-old wild type female mice, MF1 strain, housed in a 12h:12h light:dark regime before and during infection. We infected mice intraperitoneally with 1×10 ^6^ *P. chabaudi* genotype DK infected red blood cells at ring stage and terminally bled them on day 6 post infection, when the parasitaemia was around 15%. Mice were bled at ZT4, when parasites were late rings/ early trophozoites (see Prior *et al.*, 2018). Approximately 1 ml of blood was collected from each mouse, which was then split equally across cultures in all five treatment groups.

***Culturing*.** We washed infected blood twice with buffered RPMI containing no amino acids (following Spence *et al.*, 2011), before being reconstituted in the RPMI medium corresponding to each treatment (containing isoleucine, or not) ( dx.doi.org/10.17504/protocols.io.buuqnwvw). We cultured parasites in 96-well round bottom plates with total culture volumes of 200–250 µl, at ~3% haematocrit and kept the culture plates inside a gas chamber which was gassed upon closing with 88% nitrogen 7% carbon dioxide and 5% oxygen, and then placed inside a 37°C incubator. The culture medium was custom made RPMI from Cell Culture Technologies, Switzerland.

***Sampling*.** We sampled parasites in the first experiment (comparing IDC completion in isoleucine rich versus isoleucine free media) at 13–14, 16–18, 20–21, 24–26 and 27 hours after culture initiation. We also sampled parasites that had been isoleucine deprived for seven, 14 or 18 hours at six and nine hours after isoleucine addition. Samples consisted of a thin blood smear from each culture fixed with methanol and Giemsa stained. We measured the proportion of parasites in the schizont stage (as an indicator of parasites having completed their IDC) by counting ~300 parasites per blood smear. Smears were read without knowledge of the treatment group, and excluded where smear quality was too poor to count RBCs.

### Statistical analysis

We defined daily rhythmicity in the concentrations of the 134 metabolites detected by the UPLC/MS-MS platform as the detection of rhythmicity in at least two of the following circadian analysis programmes: ECHO, CircWave and JTK_Cycle. For metabolites not found to be rhythmic by at least two of the circadian analysis programmes, we also carried out Analysis of Variance to identify metabolites that varied across the day but without a detectable 24-hour rhythm ( Table 3 for breakdown of which candidate metabolites were rhythmic in each programme). To calculate the acrophase (timing of peak), we used linear mixed-effects regression models containing sine and cosine terms on all metabolites that varied across the day. Metabolites whose acrophase fell in the same 12-hour feeding or fasting window (ZT0-12 or ZT12-24) in LF and TRF infections but fell in the opposite window for the DF group were shortlisted as potential candidates to connect host feeding rhythms and the IDC schedule. We compared schizont proportion and the density of combined IDC stages using linear regression models. To avoid overfitting due to small sample sizes, “Akaike information criterion-corrected” (AICc) values were calculated for each model, and a change in two AICc (ΔAICc = 2) was chosen to select the most parsimonious model ( Brewer *et al.*, 2016).

An earlier version of this article can be found on bioRxiv (doi: https://doi.org/10.1101/2020.08.24.264689).

## Data availability

### Underlying data

Figshare: Plasmodium chabaudi genotype DK parasite stages during time series. https://doi.org/10.6084/m9.figshare.14842695.v1 ( Prior *et al.*, 2021a).

Figshare: Targeted metabolomics on malaria-infected mouse blood. https://doi.org/10.6084/m9.figshare.14842638.v1 ( Prior *et al.*, 2021b).

Figshare: Plasmodium schizont proportion in culture grown with and without isoleucine. https://doi.org/10.6084/m9.figshare.14842713.v1 ( Prior *et al.*, 2021c).

Figshare: Plasmodium schizont densities in culture grown with and without isoleucine, as well as isoleucine starvation for different durations of time. https://doi.org/10.6084/m9.figshare.14842716.v1 ( Prior *et al.*, 2021d).

Figshare: Blood glucose concentration time series in mice infected with malaria parasites https://doi.org/10.6084/m9.figshare.14912709.v1 ( Prior *et al.*, 2021e).

### Reporting guidelines

Figshare: ARRIVE checklist for “Synchrony between daily rhythms of malaria parasites and hosts is driven by an essential amino acid”. https://doi.org/10.6084/m9.figshare.14912703.v1 ( Prior *et al.*, 2021f).

Data are available under the terms of the Creative Commons Attribution 4.0 International license (CC-BY 4.0).
