# Supplementary material for: Synchrony between daily rhythms of malaria parasites and hosts is driven by an essential amino acid
Source: Wellcome Open Res. 2021 Oct 20;6:186. Originally published 2021 Jul 22. [Version 2] doi: 10.12688/wellcomeopenres.16894.2 (PMC8577053; doi:10.12688/wellcomeopenres.16894.2)
Supplement: Peer review history

# Peer review: Reviewer response for version 2

**DOI:** 10.21956/wellcomeopenres.19116.r46564

**Published:** 2021-11-08

**Authors:** Luis Larrondo

**Affiliations:** 1 ANID - Millennium Science Initiative, Millennium Institute for Integrative Biology (iBIO), Santiago, Chile; 2 Pontificia Universidad Católica de Chile, Santiago, Chile

Thanks for the detailed answers and clarifications to my comments and inquires. I have no further comments and consider this MS approved without reservations.

Is the work clearly and accurately presented and does it cite the current literature?

Yes

If applicable, is the statistical analysis and its interpretation appropriate?

Yes

Are all the source data underlying the results available to ensure full reproducibility?

Yes

Is the study design appropriate and is the work technically sound?

Yes

Are the conclusions drawn adequately supported by the results?

Partly

Are sufficient details of methods and analysis provided to allow replication by others?

Yes

Reviewer Expertise:

chronobiology, molecular genetics

I confirm that I have read this submission and believe that I have an appropriate level of expertise to confirm that it is of an acceptable scientific standard.

# Peer review: Reviewer response for version 1

**DOI:** 10.21956/wellcomeopenres.18637.r45928

**Published:** 2021-10-06

**Authors:** Xiaodong Zhuang

**Affiliations:** 1 Nuffield Department of Medicine, University of Oxford, Oxford, UK

Prior *et al. *performed an interesting study that identified a key nutrient mediator responsible for the synchrony between plasmodium blood-stage replication and the host feeding-fasting rhythm. In this study, circadian entrained wild type and circadian defect per1/2 knockout mice with different feeding schedules were infected with *P. chabaudi* followed by metabolomics analysis of blood samples at 2h intervals. The data suggest the feeding rhythm alone can override the host clock defect to regulate the rhythmic intraerythrocytic development cycle (IDC) and metabolites. Isoleucine was identified as the ‘circadian gate’ for parasites to set their time-of-day schedule for replication. Their findings provide important evidence to increase our understanding of how parasites align their lifecycle with the host's daily rhythm. Future mechanistic studies to define the role of isoleucine in regulating rhythmic IDCs are certainly warranted. The manuscript is nicely written and the results clearly demonstrated, which supports their major conclusions.

**Specific comments:**

- Abstract - Define TTFL.
- Figure 1 - Legend (B) "The experimental…".
- Figure 3 - Please label the top right and bottom left boxes.
- Figure 5 - Recommend to show ALF as a negative control.
- Figure 6c - Please provide slopes in text or normalized data to allow comparison.
- Figure 8 - Bar ‘I’ is blue for both human and mouse.
- Figure 9 - This figure is less informative; suggest move to supplementary material.
- Table 2 - It would be helpful to explain the rationale of block sampling for the metabolomics study but not for the glucose experiment.
- The page layout can be improved by re-sizing figures (for example fig.2, 3, 4) and moving some long tables (table 5 and 6) to supplementary materials.

Is the work clearly and accurately presented and does it cite the current literature?

Yes

If applicable, is the statistical analysis and its interpretation appropriate?

Yes

Are all the source data underlying the results available to ensure full reproducibility?

Yes

Is the study design appropriate and is the work technically sound?

Yes

Are the conclusions drawn adequately supported by the results?

Yes

Are sufficient details of methods and analysis provided to allow replication by others?

Yes

Reviewer Expertise:

Infectious disease; Circadian biology; Immunology

I confirm that I have read this submission and believe that I have an appropriate level of expertise to confirm that it is of an acceptable scientific standard.

# Response

**Published:** 2021-10-08

**Authors:** Kimberley Prior

**Affiliations:** University of Edinburgh, UK

We are very grateful to Reviewer 2, Xiaodong (Alan) Zhuang, for making such constructive comments and we have revised the manuscript as suggested, detailed in the following responses.

 Specific comments:

 1) Abstract - Define TTFL.

 RESPONSE: Now included.

 2) Figure 1 - Legend (B) "The experimental…".

 RESPONSE: Now fixed.

 3) Figure 3 - Please label the top right and bottom left boxes.

 RESPONSE: We removed the background colours from these portions of the Fig (in response to Reviewer 1 and explained what these regions are in the legend

 4) Figure 5 - Recommend to show ALF as a negative control.

 RESPONSE: Great idea, because the best fitting pattern for this group is linear, so we have indicated intercept in the legend and on the revised figure (also, this has become Figure 6).

 5) Figure 6c - Please provide slopes in text or normalized data to allow comparison.

 RESPONSE: This now Figure 7. The statistical analysis revealing a significant interaction confirms the slopes are different and the aim is to simply compare the groups rather than parameterise developmental patterns

 6) Figure 8 - Bar ‘I’ is blue for both human and mouse.

 RESPONSE: Thanks for spotting that – now fixed

 7) Figure 9 - This figure is less informative; suggest move to supplementary material.

 RESPONSE: We agree but the journal no long supports supplementary material so our understanding is that has be included in the vicinity of where it is cited.

 8) Table 2 - It would be helpful to explain the rationale of block sampling for the metabolomics study but not for the glucose experiment.

 RESPONSE: We’ve included a pointer to the legend to where this is explained in the methods section.

 9) The page layout can be improved by re-sizing figures (for example fig.2, 3, 4) and moving some long tables (table 5 and 6) to supplementary materials.

 RESPONSE: We agree but our understanding is that the location and size of elements is determined by the journal and outwith author’s control.

# Peer review: Reviewer response for version 1

**DOI:** 10.21956/wellcomeopenres.18637.r45125

**Published:** 2021-08-25

**Authors:** Luis Larrondo

**Affiliations:** 1 ANID - Millennium Science Initiative, Millennium Institute for Integrative Biology (iBIO), Santiago, Chile; 2 Pontificia Universidad Católica de Chile, Santiago, Chile

The work by Prior *et al.*, is a nicely written MS that reports extremely interesting data on how malaria parasites may be sensing and responding to circulating metabolic host cues to synchronize their biology. Surprisingly, under time restricted feeding protocol the synchronizing cue is not glucose levels (as it could have been suspected from other studies) but instead, is isoleucine. Importantly, this appears to be an active strategy by *Plasmodium *as opposed to a constraint imposed by host rhythms, as isoleucine deprivation does not appear to have a major cost compared to other essential nutrients. While the MS does not go into the mechanistic details on how the sensing of isoleucine occurs or on how it gates rhythmic asexual development, it does describe an important set of original and valuable observations that will help to advance the field.

                                          

 Something that needs to be clarified for all readers (without having to go back to prior publications) is why this particular experimental design was selected. In particular, one would have expected the experimental groups to also include WT in constant darkness both under ALF and TRF conditions. Yet, the only animals kept under DD were mice devoid of a functional clock (per1/2 DKO). While one could try to interpret the logic based on O’Donnell *et al.*, 2020, it is crucial for general readers to clearly grasp the underlying reasons of the design (i.e why there was no ALF for WT animals). This information is important since other reports (Rijo-Ferreira *et al.*, 2020) have shown that the parasite rhythms persist even under ad libitum conditions in WT animals (something that was not observed in O’Donnell *et al.*, 2020) yet, was such condition (WT ad libitum feeding) was not analyzed in the current MS. In addition, the data by Rijo-Ferreira *et al.*, 2020 show that in arrhythmic animals the parasite rhythms persist for about 4 days before becoming arrhythmic.

 Hirako *et al.* found that rhythmic oscillation in blood glucose levels was associated with *P. chabaudi *cell cycle synchronization. Yet, in light of the results of Prior *et al.*, it appears that this would be an association and not causation. Nevertheless, the same study by Hirako *et al.* implicated TNFR in this mechanism, and sure enough ^−/−^ animals showed no rhythms in the parasite proliferation. These observations should be further discussed.  

 Fig 1, legend: “cycles of 12-hours with food followed by 12 h without access to food (TRF, time-restricted feeding”.

 To use TRF only here is somehow weird as it is equivalent to the other protocols (light or dark feeding). Thus, the other protocols are also equivalent to TRF. Indeed, in several of the papers cited in the MS (as well as in many other publications) what is presented here as light or dark feeding would be called TRF.

 Figur3 3B: Gray color is not explained

 “Mean blood-glucose concentration differed between the groups, being higher in DF and TRF mice (DF=8.55±0.14 mmol/L, TRF=8.59±0.13 mmol/L)”

 These numbers are hard to recognize in the data (Fig 4), where the lines and shading are mean ± SEM glucose concentrations. Indeed, in most cases, the SEM in the plots span at least 1 unit (quite different from the +/- 0.1 units referred to in the text). Are those glucose concentration average values, or values at a particular moment?   

 “Isoleucine cannot be perturbed in vivo without causing confounding off-target effects on the host or interference by host homeostasis”.

 References and a brief explanation are needed.

 Fig 8. The orange bar for I (human) is missing and instead, the blue bar (rodent) has twice the width. Since Ile is absent from the human hemoglobin it should be replaced by a Y -axis 0 orange bar, instead of being replaced by a broader blue one.

 Fig 9 contains important data, although it is plotted in a way that is not useful. Each of the columns in the x axis should have a number that allows identifying (from the legend) the corresponding metabolite. Likewise, changing the size of each sphere (dot) would allow better visual resolution between one set and the other

 Ideally one would like to see experiments that would compare the qualitative effect of diets on the parasite rhythms. In particular, it would be interesting to compare existing feeding paradigms (i.e high fat diet, TRF vs *ad libitum*) versus high protein diets (TRF vs *ad libitum*). Yet, conducting and analyzing these experiments would be another paper in itself. Nevertheless, there may be existing literature that allows establishing interesting correlations between protein (Ile) content and the characteristics of the disease. Likewise, one would expect that this effect would be clearer in humans, particularly in cases where dietary interventions/ food supplements modify isoleucine circulating levels. While such association would establish only correlation (and not causation), it would enrich the discussion.   

 “Wild type C57BL/6 mice were housed” Specify sex of the animals.

Is the work clearly and accurately presented and does it cite the current literature?

Yes

If applicable, is the statistical analysis and its interpretation appropriate?

Yes

Are all the source data underlying the results available to ensure full reproducibility?

Yes

Is the study design appropriate and is the work technically sound?

Yes

Are the conclusions drawn adequately supported by the results?

Partly

Are sufficient details of methods and analysis provided to allow replication by others?

Yes

Reviewer Expertise:

chronobiology, molecular genetics

I confirm that I have read this submission and believe that I have an appropriate level of expertise to confirm that it is of an acceptable scientific standard, however I have significant reservations, as outlined above.

# Response

**Published:** 2021-10-09

**Authors:** Kimberley Prior

**Affiliations:** University of Edinburgh, UK

We are very grateful to Reviewer 1, Luis Larrondo, for making such constructive comments and we have revised the manuscript as suggested, detailed in the following responses.

 COMMENT 1) Something that needs to be clarified for all readers (without having to go back to prior publications) is why this particular experimental design was selected. In particular, one would have expected the experimental groups to also include WT in constant darkness both under ALF and TRF conditions. Yet, the only animals kept under DD were mice devoid of a functional clock (per1/2 DKO). While one could try to interpret the logic based on O’Donnell et al., 2020, it is crucial for general readers to clearly grasp the underlying reasons of the design (i.e why there was no ALF for WT animals). This information is important since other reports (Rijo-Ferreira et al., 2020) have shown that the parasite rhythms persist even under ad libitum conditions in WT animals (something that was not observed in O’Donnell et al., 2020) yet, was such condition (WT ad libitum feeding) was not analyzed in the current MS. In addition, the data by Rijo-Ferreira et al., 2020 show that in arrhythmic animals the parasite rhythms persist for about 4 days before becoming arrhythmic.

*RESPONSE: As noted, other studied have focused on asking what IDC schedules occur under different combinations of lightdark cycles, feeding-fasting treatments and host TTFL manipulations. Rijo-Ferreira et al., 2020 reveals that the process simply of food intake does not schedule the IDC, thus O’Donnell et al., 2020 and others (Hirako et al., 2018, Prior et al., 2018) are best interpreted as finding process(es) involved in food digestion/fasting schedule the IDC. The aim of the present study was to leverage these observations – which have proved largely robust across studies - to compare and contrast treatments in which different IDC schedules occur. As such, a fully factorial design including WT mice in DD (whether TRF or ALF) would not provide a different IDC schedule to the treatments we selected. This is because (i) the IDC schedule is not directly scheduled by LD cycles (Prior et al., 2018, Hirako et al., 2018); (ii) WT mice in DD would free run (with a period very close to 24 hr in this strain (Schwartz and Zimmerman, 1990)) and maintain the same feeding rhythms under ALF as WT ALF mice in LD cycles (O’Donnell et al., 2020); (iii) if we applied TRF to WT mice in DD in subjective day, this would mirror the conditions in WT LF mice; and (iv) we focused on using TRF treatments (i.e. light-fed, LF, and dark-fed, DF) for the WT mice to avoid the potentially confounding effects of ALF mice being able to take in more food across the circadian cycle on metabolite concentrations. Further, adding more treatment groups would have traded-off against the number of replicates within treatments, due to the time constraints on completing sample collection within an appropriate window. We have added an explanation for the design at the start of the results section.*

 COMMENT 2) Hirako et al. found that rhythmic oscillation in blood glucose levels was associated with P. chabaudi cell cycle synchronization. Yet, in light of the results of Prior et al., it appears that this would be an association and not causation. Nevertheless, the same study by Hirako et al. implicated TNFR in this mechanism, and sure enough -/- animals showed no rhythms in the parasite proliferation. These observations should be further discussed.

*RESPONSE: The 3rd paragraph of the discussion explores how the results of Hirako et al. and indeed our previous work (Prior et al., 2018) can be reconciled with our findings about isoleucine. For example, glucose is essential for isoleucine uptake (Martin & Kirk, 2007), and high blood glucose concentrations of isoleucine are associated with uptake of glucose by host cells, including by TNF stimulated immune cells (Elased & Playfair, 1994; Hirako et al., 2018). We have altered this paragraph to conclude that whilst isoleucine appears to be the main driver of the IDC schedule, glucose likely mediates its impact via multiple, non-mutually exclusive, activities. Further, we have now included another experiment and new Figure providing more evidence that glucose is not a direct driver of the IDC schedule.*

 COMMENT 3) Fig 1, legend: “cycles of 12-hours with food followed by 12 h without access to food (TRF, time- restricted feeding”. To use TRF only here is somehow weird as it is equivalent to the other protocols (light or dark feeding). Thus, the other protocols are also equivalent to TRF. Indeed, in several of the papers cited in the MS (as well as in many other publications) what is presented here as light or dark feeding would be called TRF.

*RESPONSE: Good point! We have clarified that these are all forms of TRF but to keep the acronyms of the treatment groups easy to follow we have retained our nomenclature.*

 COMMENT 4) Figur3 3B: Gray color is not explained

*RESPONSE: This was just a background colour. To avoid confusion we have changed this to white.*

 COMMENT 5) “Mean blood-glucose concentration differed between the groups, being higher in DF and TRF mice (DF=8.55±0.14 mmol/L, TRF=8.59±0.13 mmol/L)” These numbers are hard to recognize in the data (Fig 4), where the lines and shading are mean ± SEM glucose concentrations. Indeed, in most cases, the SEM in the plots span at least 1 unit (quite different from the +/- 0.1 units referred to in the text). Are those glucose concentration average values, or values at a particular moment?

*RESPONSE: We quoted the overall means ±SEM of the time series (i.e. intercepts) in the text and have clarified this. We note that visual inspection of Fig4 suggests the mean patterns for DF and TRF exceed 8mmol/L for most of the sampling window, unlike the LF and AL groups.*

 COMMENT 6) “Isoleucine cannot be perturbed in vivo without causing confounding off-target effects on the host or interference by host homeostasis”. References and a brief explanation are needed.

*RESPONSE: We searched the literature for approaches to perturbing blood-isoleucine concentrations in mice but were unrewarded because there are several issues to overcome. Isoleucine is essential for mice as well as parasites, so hosts cannot be maintained on isoleucine-free food without negative health consequences, particularly via its roles in the immune system (Rivas- Santiago et al, 2011). For example, even short-term limitation of isoleucine to 50% of optimal for 3 weeks results in altered immune performance (Mao et al, 2018), including a reduction in complement making it difficult to separate the consequences isoleucine perturbations from depressed innate immune defences that are crucial in the acute phase of malaria infection. Isoleucine also modulates VEGF expression which is involved in recovery from anaemia (Murata et al, 2007), a hallmark of malaria infection. Thus, an in vitro system allows far more precise control over isoleucine levels and the timing of its availability to parasites for our proof of principle experiments. We have now summarised this in the discussion.*

 COMMENT 7) Fig 8. The orange bar for I (human) is missing and instead, the blue bar (rodent) has twice the width. Since Ile is absent from the human hemoglobin it should be replaced by a Y -axis 0 orange bar, instead of being replaced by a broader blue one.

*RESPONSE: Thanks for spotting this “graphical typo”. Now corrected*

 COMMENT 8) Fig 9 contains important data, although it is plotted in a way that is not useful. Each of the columns in the x axis should have a number that allows identifying (from the legend) the corresponding metabolite. Likewise, changing the size of each sphere (dot) would allow better visual resolution between one set and the other

*RESPONSE: Apologies for the confusion. We have added numbers to the x axis that correspond to a metabolite numbering system added to the legend. We have now plotted the median concentration for each treatment for every metabolite, instead of plotting the raw data. As this figure is not about the metabolite dynamics, but about which metabolites remain in our analysis, this results in 4 data points per metabolite and a much simpler figure.*

 COMMENT 9) Ideally one would like to see experiments that would compare the qualitative effect of diets on the parasite rhythms. In particular, it would be interesting to compare existing feeding paradigms (i.e high fat diet, TRF vs ad libitum) versus high protein diets (TRF vs ad libitum). Yet, conducting and analyzing these experiments would be another paper in itself. Nevertheless, there may be existing literature that allows establishing interesting correlations between protein (Ile) content and the characteristics of the disease. Likewise, one would expect that this effect would be clearer in humans, particularly in cases where dietary interventions/ food supplements modify isoleucine circulating levels. While such association would establish only correlation (and not causation), it would enrich the discussion.

*RESPONSE: We agree these are all interesting avenues for future work – particularly, by comparing and contrasting parasite gene expression patterns across multiple feeding paradigms, insight into the parasite’s time keeping mechanism might be revealed. Given that malaria is almost exclusively a disease in LMICs and that controlled human infection trails do not consider dietary details, this kind of information is not available to discuss.*

 COMMENT 10) “Wild type C57BL/6 mice were housed” Specify sex of the animals.

*RESPONSE: Sexes were already specified in the detailed descriptions of each experiment, in the methods section*

 REFERENCES

 Elased K, Playfair JH: Hypoglycemia and hyperinsulinemia in rodent models of severe malaria infection. Infect Immun. 1994;62(11):5157–5160. 7927799 10.1128/iai.62.11.5157-5160.1994 303239

 Hirako IC, Assis PA, Hojo-Souza NS, et al.: Daily Rhythms of TNFα Expression and Food Intake Regulate Synchrony of Plasmodium Stages with the Host Circadian Cycle. Cell Host Microbe. 2018;23(6):796–808.e6. 29805094

 Mao et al. 2018. l-Isoleucine Administration Alleviates Rotavirus Infection and Immune Response in the Weaned Piglet Model. Front. Immunol., 16 July 2018. PMCID: PMC6054962

 Martin RE, Kirk K: Transport of the essential nutrient isoleucine in human erythrocytes infected with the malaria parasite Plasmodium falciparum. Blood. 2007;109(5):2217–2224. 17047158 10.1182/blood-2005-11-026963

 Murata et al. 2007. Isoleucine, an Essential Amino Acid, Prevents Liver Metastases of Colon Cancer by Antiangiogenesis. Cancer Res 2007 Apr 1;67(7):3263-8. PMID: 17409434

 O’Donnell AJ, Prior KF, Reece SE: Host circadian clocks do not set the schedule for the within-host replication of malaria parasites. Proc Biol Sci. 2020;287(1932):20200347. 32781954 10.1098/rspb.2020.0347 7575513

 Prior KF, van der Veen DR, O’Donnell AJ, et al.: Timing of host feeding drives rhythms in parasite replication. PLoS Pathog. 2018;14(2):e1006900. 29481559 10.1371/journal.ppat.1006900 5843352

 Rijo-Ferreira F, Acosta-Rodriguez VA, Abel JH, et al.: The malaria parasite has an intrinsic clock. Science. 2020;368(6492):746– 753. 32409471 10.1126/science.aba2658 7409452

 Rivas-Santiago et al. 2011. Induction of β-defensins by l-isoleucine as novel immunotherapy in experimental murine tuberculosis. Clin Exp Immunol. 2011 Apr;164(1):80-9. PMCID: PMC3074220

 Schwartz, W, Zimmerman, P. Circadian timekeeping in BALB/c and C57BL/6 inbred mouse strains. The Journal of Neuroscience. 1990 Nov;10(11):3685-3694.
